# Genome-Wide Transcriptional Regulation Mediated by Biochemically Distinct SWI/SNF Complexes

**DOI:** 10.1371/journal.pgen.1005748

**Published:** 2015-12-30

**Authors:** Jesse R. Raab, Samuel Resnick, Terry Magnuson

**Affiliations:** 1 Department of Genetics, University of North Carolina at Chapel Hill, Chapel Hill, North Carolina, United States of America; 2 Lineberger Comprehensive Cancer Center, University of North Carolina at Chapel Hill, Chapel Hill, North Carolina, United States of America; University of Chicago, UNITED STATES

## Abstract

Multiple positions within the SWI/SNF chromatin remodeling complex can be filled by mutually exclusive subunits. Inclusion or exclusion of these proteins defines many unique forms of SWI/SNF and has profound functional consequences. Often this complex is studied as a single entity within a particular cell type and we understand little about the functional relationship between these biochemically distinct forms of the remodeling complex. Here we examine the functional relationships among three complex-specific ARID (AT-Rich Interacting Domain) subunits using genome-wide chromatin immunoprecipitation, transcriptome analysis, and transcription factor binding maps. We find widespread overlap in transcriptional regulation and the genomic binding of distinct SWI/SNF complexes. ARID1B and ARID2 participate in wide-spread cooperation to repress hundreds of genes. Additionally, we find numerous examples of competition between ARID1A and another ARID, and validate that gene expression changes following loss of one ARID are dependent on the function of an alternative ARID. These distinct regulatory modalities are correlated with differential occupancy by transcription factors. Together, these data suggest that distinct SWI/SNF complexes dictate gene-specific transcription through functional interactions between the different forms of the SWI/SNF complex and associated co-factors. Most genes regulated by SWI/SNF are controlled by multiple biochemically distinct forms of the complex, and the overall expression of a gene is the product of the interaction between these different SWI/SNF complexes. The three mutually exclusive ARID family members are among the most frequently mutated chromatin regulators in cancer, and understanding the functional interactions and their role in transcriptional regulation provides an important foundation to understand their role in cancer.

## Author Summary

The SWI/SNF chromatin remodeling complex is made of multiple subunits, and the inclusion of different mutually exclusive subunits allows potentially many biochemically distinct forms of SWI/SNF. Members of this complex are among the most frequently mutated genes in cancer. Here, we present the first genome-wide localization maps of three mutually exclusive ARID subunits, and identify their role in regulating transcription. The ARID subunits have largely overlapping localization, and affect transcription of many of the same genes. Within these overlapping classes we identified extensive cooperation between ARID1B and ARID2 in the repression of hundreds of genes, as well as competitive interactions between ARID1A and the other ARIDs. These distinct regulatory modalities were characterized by co-localization with specific transcription factors. Together these data provide insight into the functional relationships between multiple distinct SWI/SNF complexes. SWI/SNF controls gene expression at thousands of genes and in most of these cases multiple distinct forms of the complex interact to specify the gene’s transcription status in a particular cell. Understanding the role of each distinct form of SWI/SNF within a cell is a critical first step towards untangling the effects of SWI/SNF subunit loss in cancer.

## Introduction

The SWI/SNF (SWItch/Sucrose Non-Fermentable) family of chromatin remodeling proteins plays a critical role in developmental processes [1]. In mice and humans this complex is composed of 12–15 subunits and certain positions within the complex can be filled by distinct mutually exclusive proteins [2, 3]. The inclusion of different subunits gives rise to a functionally diverse set of complexes, capable of driving a cell into a specific lineage or critical for maintaining pluripotency [4–6]. There are a large number of possible forms of the SWI/SNF complex based on the inclusion of such mutually exclusive subunits, and despite the pivotal role for many of these in development and disease the functional relationships between mutually exclusive subunits remains under-explored.

The ARID (AT-Rich Interacting Domain) domain containing proteins of the SWI/SNF complex are examples of three mutually exclusive subunits, and their inclusion distinguishes three biochemically distinct forms of SWI/SNF. ARID1A and ARID1B are found in the BAF-A and BAF-B (Brahma Associated Factor) complex respectively, while ARID2 is found in the PBAF (Polybromo BAF) complex. The functional interactions between distinct forms of this heterogeneous remodeling complex have not been studied genome-wide. All three ARID genes are mutated in a wide variety of tumors [7–15]. Of particular interest to our study, all three are recurrently mutated in hepatocellular carcinoma and mutations are mutually exclusive suggesting they function through related mechanisms [10–12]. A current focus of therapeutically targeting SWI/SNF mutant tumors is based on findings of synthetic lethality between subunits, yet we currently understand little about how these subunits functionally interact [16, 17].

In this paper we examine two questions related to the functional overlap of distinct forms of the SWI/SNF remodeling complex. First, do different SWI/SNF complexes extensively cooperate or compete to regulate transcription? Second, do distinct forms of the SWI/SNF complex interact with unique or common cis-regulatory modules to mediate their role in transcription? To address these questions we performed ChIP-seq and RNA-seq in HepG2 cells, a human liver cancer cell line for which extensive transcription factor binding data are available from the ENCODE project [18].

We present evidence that ARID1A, ARID1B, and ARID2 have a highly overlapping role in transcriptional regulation in HepG2 cells. Additionally, we provide the first genome-wide mapping of biochemically distinct SWI/SNF complexes and show a high degree of overlap between binding sites for the different ARID proteins. We identify putative regulatory modules specific for the different types of ARID mediated regulation. Using RNAi against combinations of ARIDs we show ARID1A and ARID2 have frequent competitive interactions, while ARID1B and ARID2 are both required for repression and are not redundant. Our study provides insight into how distinct forms of the SWI/SNF complex regulate transcription in an interdependent manner and provides mechanistic insight into recent observations of synthetic lethal interactions between SWI/SNF subunits [16, 17, 19].

## Results

### Identification of genes regulated by ARID proteins

All three ARID members of the SWI/SNF complex are expressed in the human liver cancer cell line HepG2, and in hepatocellular carcinoma all three ARIDs are recurrently mutated in a mutually exclusive fashion [10–12]. ARID1A is expressed at the highest level in most cell types including HepG2, while ARID2 is expressed at a lower level (S1 Fig, ARID1A = 18.9 TPM (transcripts per million), ARID1B = 13.4 TPM, ARID2 = 8.3 TPM in ENCODE data [18], S1 Fig) Using RNAi we depleted each of the ARID subunits and asked whether they controlled unique transcriptional programs. We were able to reduce the expression levels of all three ARID genes to 25–50% of normal levels (S2A and S2B Fig). The moderate level of knockdown achieved and the short duration of the experiment (72 hours) may allow cells to remain healthy while still allowing the detection of gene expression changes. We identified 284, 1289, and 2682 differentially expressed genes following ARID1A, ARID1B, and ARID2 knockdown at a FDR of 0.1 for ARID1A, and 0.05 for ARID1B and ARID2. We validated expression changes using a second set of siRNAs for selected genes using qPCR (S3A–S3C Fig). ARID1A and ARID2 loss caused similar proportions of genes to be up-regulated compared to down-regulated (116 up-regulated, 168 down-regulated ARID1A; 1459 up-regulated, 1223 down-regulated ARID2). However, ARID1B depletion led to increased expression in more than twice as many genes as decreased expression (865 up-regulated, 424 down-downregulated), suggesting ARID1B had a more prominent role in repression in HepG2 cells.

**Fig 1:**
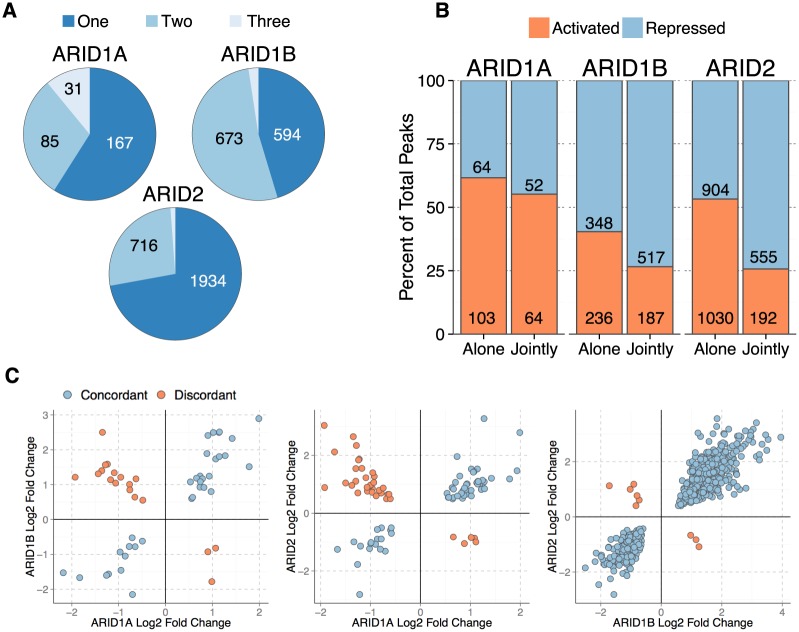
Analysis of ARID mediated transcriptional regulation. A. Number of genes altered by loss of an ARID that are affected by one, two, or three ARIDs. B. Direction (Activated or Repressed) of gene expression changes in alone compared to jointly regulated genes (ARID1A—Not Significant, ARID1B—p-value = 1.9 × 10^−7^, ARID2—p-value = 1.64 × 10^−37^, Chi-squared test). C. Pair-wise comparison of Log2 Fold Changes following knockdown of each ARID shows the numbers of genes regulated in the same direction (concordant—blue) compared to regulated in an opposing manner (discordant—pink). Positive values for log2 fold change indicate repressive action, while negative log2 fold change values indicate activation. ARID1A—ARID1B r^2^ = 0.185, p-value = 0.001; ARID1A-ARID2 r^2^ = 0.03, p-value = 0.054; ARID1B-ARID2 r^2^ = 0.85, p-value <2.2 × 10^−16^)

Given previous examples in the literature of competitive interactions between ARID family members in different developmental contexts [4, 5, 20, 21], we hypothesized that ARID proteins would regulate overlapping gene sets. We calculated the fraction of genes altered by one, two, or three ARID family members. This revealed that 41% (ARID1A), 54%(ARID1B), and 27% (ARID2) of genes were affected by the loss of more than one ARID (Fig 1A), an overlap that is significant for each of the three ARIDs (ARID1A 2.6 × 10^−12^, ARID1B <2.2 × 10^−16^, ARID2 <2.2 × 10^−16^; hypergeometric test). In the remainder of the manuscript we refer to genes transcriptionally regulated by a single ARID as ‘alone’ and those regulated by more than one ARIDs as ‘jointly’ (S10 Table). We performed pathway analysis on the alone and jointly regulated gene sets for each ARID (S7, S8 and S9 Tables). ARID1A alone genes were enriched for numerous gene sets involved with liver cancer and liver development. While ARID1B and ARID2 joint categories both were enriched for HDAC target genes and SMAD signaling.

In total, loss of an ARID protein affected the expression of 3453 unique genes of the total 17157 genes expressed at a level detected in our data set (20.1%). Of these 3453 genes 747 were affected by multiple ARIDs (21.6%). Together, these data suggest an extensive role for functional interaction among biochemically discrete forms of SWI/SNF.

### ARID proteins regulate gene expression in both a concordant and discordant manner

We next compared the direction of gene expression changes of genes regulated alone with genes regulated jointly (Fig 1B). While ARID1A regulated roughly an equal number of genes positively and negatively in the alone and joint categories, ARID1B and ARID2 loss caused distinct effects at genes regulated by multiple ARIDs. When ARID2 is the only Arid affecting expression of a gene, it is equally likely to repress a gene as it is to activate a gene. However, when ARID2 and another ARID regulate the same gene, ARID2 is twice as likely to repress the target gene (Chi-squared p-value 1.64 × 10^−37^). While ARID1B primarily represses genes, it also is significantly more likely to repress transcription when functioning with another Arid (Fig 1B, Chi-squared p-value 1.9 × 10^−7^).

If ARID subunits are mainly functioning in opposition in HepG2 cells we would expect them to have discordant effects on gene expression. We compared changes in expression for pairs of ARID proteins at jointly regulated genes. Those genes where both ARIDs caused an expression change in the same direction are said to be concordant, while those regulating expression in opposite directions are called discordant. From this analysis we cannot conclude the complexes directly compete or cooperate, but instead use these terms in relation to the functional consequence of the dual regulation by a pair of ARID proteins. When ARID1B or ARID2 were regulating genes in conjunction with ARID1A there we observed both concordant and discordant changes (ARID1A-ARID1B r^2^ = 0.185, ARID1A-ARID2 r^2^ 0.03, Fig 1C).

However, when ARID1B and ARID2 regulated the same gene there was an extreme degree of concordance (Fig 1C, r^2^ = 0.85, p-value <2.2 × 10^−16^). The majority of these interactions consisted of a requirement for both ARID1B and ARID2 to repress gene expression. Together these results suggest that ARID1B and ARID2 regulate a highly similar transcriptional network.

### ARIDs bind at active genomic regions

Given the high degree of overlap in the regulation of gene expression by each ARID, we hypothesized there would be extensive overlap in the binding sites for the ARID subunits. Widespread genomic co-localization of distinct families of chromatin remodelers has been observed in mouse mammary epithelial cells [22], suggesting co-occupancy of chromatin remodeling complexes may be a general feature of transcriptional control mediated by chromatin remodeling complexes. We used ChIP-seq to identify the binding sites of ARID1A, ARID1B, and ARID2. We also mapped the genomic location of BAF47 (SNF5/INI1/SMARCB1), a core SWI/SNF member previously mapped by ChIP-seq in HeLa cells [23]. We identified 12852 ARID1A, 8728 ARID1B, 12995 ARID2, and 20718 BAF47 peaks (S1, S2, S3 and S4 Tables). Each ARID subunit individually had high overlap with BAF47 bound regions (63–72%, S4A–S4C Fig). The union of all ARID bound regions overlapped approximately 73% of BAF47 bound regions in our dataset (S4D Fig). Additionally, we compared BAF47 bound regions to BAF47 binding sites identified by Euskirchen et al. and found 80% of the peaks identified in that dataset were also found in our dataset (S4E Fig). Together this suggests we identified a large number of the true binding sites for these factors in HepG2 cells.

**Fig 2:**
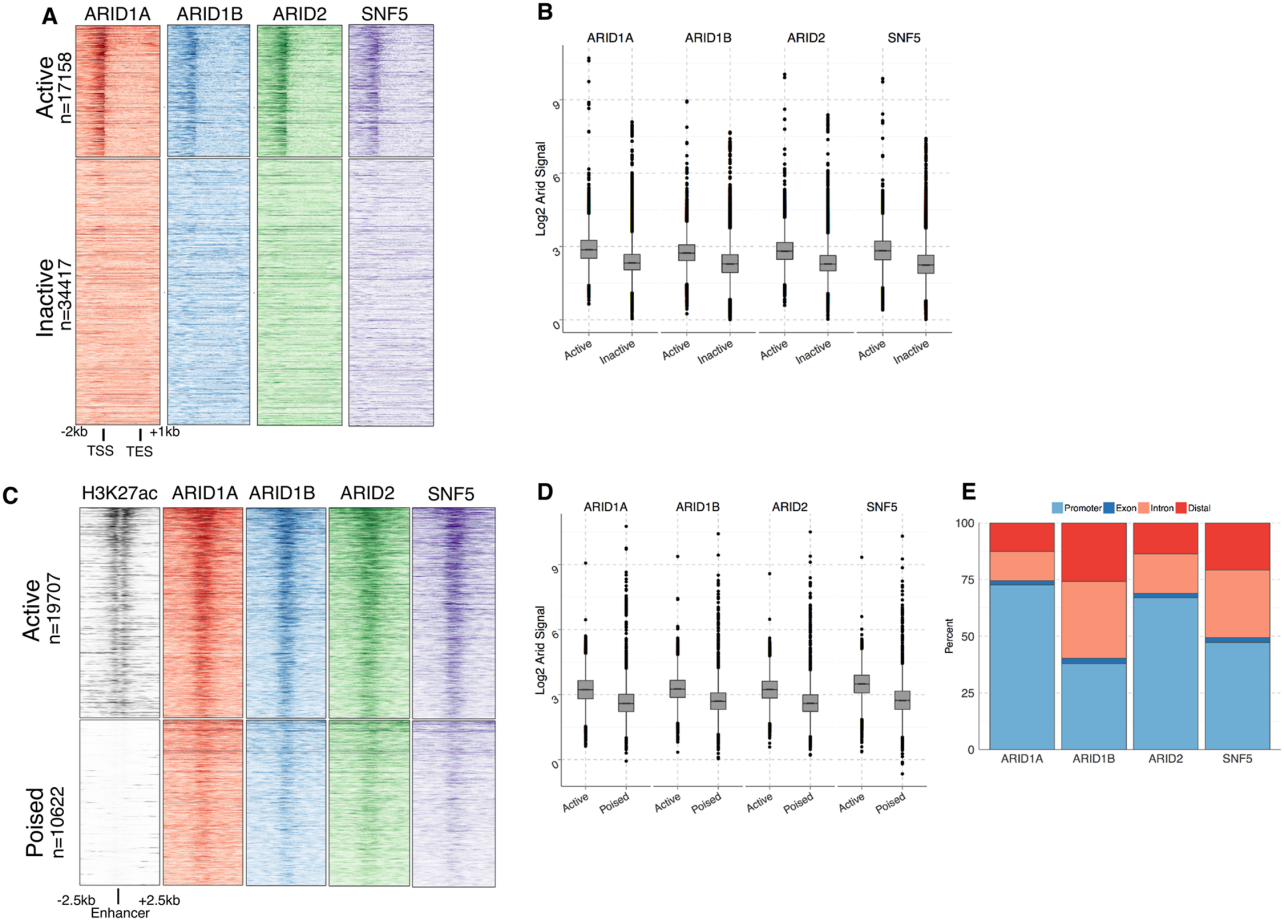
Identification of ARID bound regions A. ChIP-seq signal aligned to all transcripts (Gencode annotations V16). Rows in top panel correspond to 17158 expressed transcripts from RNA-seq data, ordered by relative expression level. Bottom panel is all remaining non-expressed/lowly expressed transcripts unordered. B. Signal of each ARID at the different classes of transcription start sites (quantification of panel A). C. ChIP-seq signal aligned to all enhancers defined using ENCODE data for p300 and H3K4me1. Enhancers are greater than 2kb from the nearest TSS and active enhancers (top panels—19707 enhancers) are distinguished from poised enhancers (bottom panel—10622 enhancers) by presence of H3K27ac. D. Signal of each ARID at active compared to poised enhancers (quantification of panel C). E. Location of ARID bound regions relative to genomic features.

We then compared ARID binding at all transcription units in HepG2 cells (Fig 2A). All three ARIDs bound near the transcription start site (TSS) of most expressed transcripts. Very little ARID binding was localized to non-expressed transcripts suggesting ARID binding correlates with active transcription (Fig 2A and 2B, Wilcoxon test p-value <2.2 × 10^−16^).

Using ENCODE data in HepG2 cells, we performed a similar analysis using all enhancers identified in HepG2 cells [18]. We defined enhancers as overlapping peaks of p300 and H3K4me1 that were greater than 2kb from the nearest TSS [18, 24]. We further subdivided these regions based on the presence (active) or absence (poised) of H3K27ac. ARID binding was evident at nearly all enhancers, with stronger signal associated with more H3K27ac, suggesting ARID binding correlated with potential enhancer activity (Fig 2C and 2D, Wilcoxon test p-value <2.2 × 10^−16^)).

Finally, we analyzed the distribution of peak locations to determine if biochemically distinct forms of SWI/SNF localized to common types of genomic features. ARID1A and ARID2 were found at similar annotations in the genome. These peaks were more skewed towards promoters and gene bodies (exons), while ARID1B bound to more distal and intronic features. Over 70% of ARID1A peaks were in promoters of genes (TSS +/- 2kb), while ARID2 peaks were slightly less localized to the promoter (60%), and ARID1B peaks were found in promoters only 25% of the time (Fig 2E). This distribution suggests ARID1B may be more commonly found at enhancers, which is reflected in the chromatin modifications present at the ARID bound regions. ARID1A and ARID2 peaks had more H3K4me3, consistent with the proximity of these peaks to promoters, while ARID1B peaks were marked by more H3K4me1 consistent with ARID1B localizing to enhancers (S5 Fig). The genomic distribution of SNF5 was intermediated to that of the ARIDs (Fig 2E).

### ARID proteins localize primarily to common genomic regions

**Fig 3:**
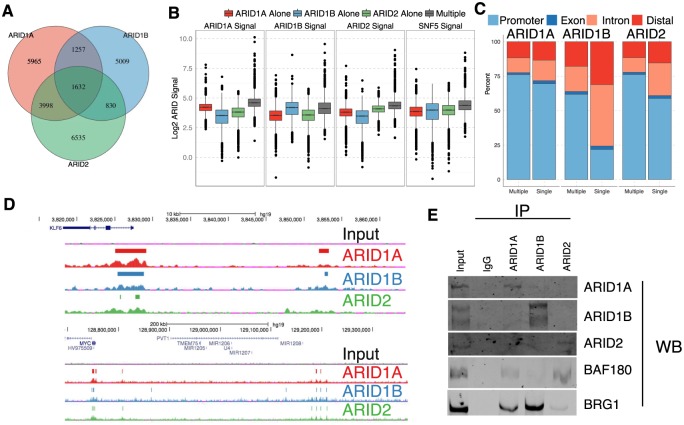
Overlap of ARID binding. A. Venn-diagram depicting overlap among peaks for each of three ARID proteins. B. Comparison of signal at ARID1A alone, ARID1B alone, ARID2 alone, and Multiple bound ARID regions. C. Genomic distribution for ARID peaks, categorized by whether the peak was bound by a single ARID or multiple ARID subunits. D. Example of ARID1A, ARID1B, and ARID2 binding at the *KLF6* (top) and *MYC* (bottom) loci. Colored bars denote called peaks for each ARID. All scales are equal, and are normalized to reads per 30bp per 10 million mapped reads. E. Co-immunoprecipitations with each ARID subunit followed by western blotting for each ARID subunit as well as BAF180 (PBAF subunit), and BRG1.

Given the high level of signal for each ARID at promoters and enhancers it is apparent that many of the ARID binding sites in the genome are shared by more than one ARID. We found approximately half of the sites for a particular ARID were bound by multiple ARIDs (ARID1A = 54%, ARID1B = 42%, ARID2 = 50% Fig 3A). However, a relatively small set of 1632 of 25226 total peaks were bound by all three ARIDs (6.5%), despite the apparently high level of signal associated with all promoters and enhancers (Fig 2A and 2B). One possibility is that lower than expected overlap between peaks is a reflection peak of calls that are overly conservative. To address this we split each set of ARID bound regions into ‘single’ and ‘multiple’ categories and calculated ChIP signal for each ARID at these peaks. This demonstrated that the ‘single’ designation is not simply a reflection of peak calling (Fig 3B, S6 Fig). For example, there was less ARID1B and ARID2 binding at ARID1A single peaks compared to ARID1A multiple peaks (S6 Fig). However, there are examples of mis-categorized loci in this analysis, likely due to the technical challenge of defining the true set of ARID bound regions. We therefore consider the ‘single’ category to be an overestimate of the true number of peaks bound only by a single ARID.

Using these categorizations we evaluated whether ‘single’ compared to ‘multiple’ bound ARID peaks localized to different types of genomic elements. ARID1A single bound regions were distributed similar to ARID1A multiple bound regions within the genome. However, for ARID1B and ARID2, binding sites in the promoters or exons of genes were more often bound by multiple ARIDS while sites located in introns or in intergenic regions were only bound by multiple ARIDS approximately 40% of the time (Fig 3C). Common and more highly occupied sites tended to be associated with promoters (Fig 3C and 3D). For example, at the *KLF6* locus a strong signal for all three ARIDs is localized in the vicinity of the *KLF6* transcription start site (TSS), while an upstream regulatory element is more variably occupied (Fig 3D). Similarly at the *MYC* locus, all three ARIDs bind near the TSS, with more variable binding at presumed enhancers distally located. For the remainder of the analysis of ARID bound regions, we separated peaks into those that were bound by ARID1A, ARID1B, or ARID2 alone and a set of ‘multiple’ peaks derived from regions bound by more than one ARID. Despite the frequent co-occurence of ARID binding, we did not detect direct physical association between the ARIDs under low stringency co-immunoprecipitations (Fig 3E). However, we detected PBRM1 (also known as BAF180) ARID1A immunoprecipitations, consistent with previous reports [25].

### ARID subunits localize with distinct chromatin regulators and transcription factors

Large groups of chromatin regulating proteins co-localize to common genomic regions [26]. Given that multiple bound sites were most commonly associated with promoters, while single bound loci were more variable in genomic location, we hypothesized that distinct forms of SWI/SNF would co-localize with transcription factors or chromatin regulators based on the type of ARID interaction. For example, ARID1A only bound regions might recruit different chromatin regulators than ARID1B only bound regions. To identify distinct co-regulatory modules for each type of ARID bound region we used ENCODE ChIP-seq data from HepG2 cells [18] (77 data sets, see S5 Table for details). Using these data sets we evaluated the enrichment for each factor and the correlation between pairs of factors at each of the four peak classes (ARID1A only, ARID1B only, ARID2 only, and multiple bound sites).

**Fig 4:**
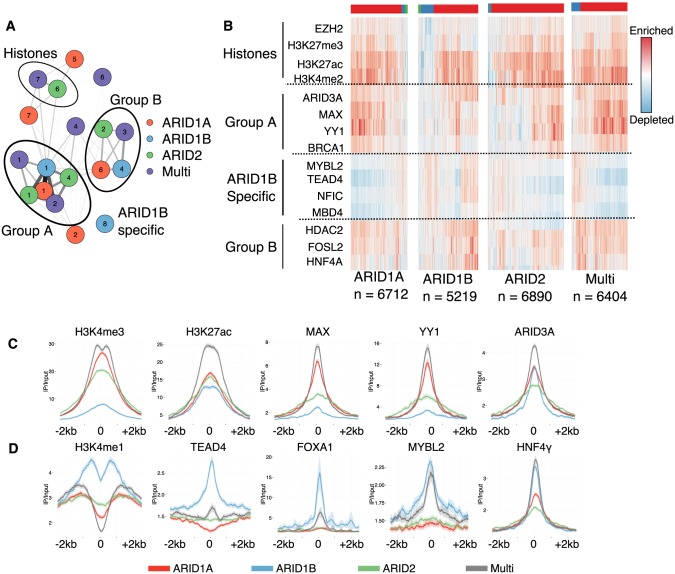
Identifcation of transcription factor clusters at ARID bound regions. A. Network diagram of similarity between cluster assignments for each ARID. Edge weight signifies the number of transcription factors or histone modifiers common to both nodes, and the color of the node signifies which ARID peak set the cluster refers to (see Materials and methods for details). Placement of nodes within the network is done using the qgraph R package and the ‘spring’ layout. B. Enrichment of transcription factors at different peak sets. Colored bar along top indicate KMeans cluster assignment of the peaks (k = 3). C. Metagene plots centered at the ARID bound peak center +/- 2.5kb for each of the ChIP-seq signals more common at ARID1A bound regions, and D. ARID1B bound regions. For all panels line represents the average signal centered on a particular class of ARID peaks, with the shading depicting the 95% confidence interval.

We clustered the ENCODE ChIP-seq data based on Spearman correlation coefficient and removed any transcription factors or histone modifications that were not enriched above a minimum level (S7A–S7D Fig). Using the clusters assigned to each factor we compared how similar any two clusters were using the number of shared factors as a measure of similarity between clusters (Fig 4A). One cluster consisted mainly of histone modifications, and these modifications were enriched highly at each of the four peak classes. Both active (H3K4me2 and H3K27ac) and inactive (H3K27me3, EZH2) modifications were shared between ARID2 and Multiple bound clusters, while these modifications were split into two separate clusters for ARID1A (Clusters 5 and 7). The same histone modifications were also enriched at ARID1B alone bound sites, but were located in a cluster containing many other transcriptional regulators. The largest group of clusters (Group A Fig 4A), contained both positive general transcription activators (MAX, TBP) as well as negative regulators (COREST, SIN3A). A second cluster of transcriptional regulators (Group B Fig 4B) is seen for all four peak classes and contains tissue-specific transcription factors such as HNF4-*α*, HNF4*γ*, and FOXA1. Within a set of peaks (ARID1A, ARID1B, ARID2, Multi) enrichment of these transcription factors was relatively consistent. We clustered peaks based on enrichment scores for several defining members of the above clusters and most peaks fell within one large cluster for each group (Fig 4B).

We also identified one cluster (ARID1B Cluster 8, Fig 4B) that shared no connections with enriched clusters at the other peaks. This cluster contained four genes (NFIC, TEAD4, MYBL2, MBD4, Fig 4B, S8 Fig). NFIC (Nuclear Factor I/C) is involved in transcriptional activation and replication. TEAD4 (TEA Domain Family Member 4) is a member of the transcriptional enhancer factor (TEF) family of proteins. It is not normally expressed in the liver, but it is up-regulated in cancer. MYBL2 (v-myb avian myeloblastosis viral oncogene homolog-like 2) is a transcription factor involved in cell cycle progression as a member of the DREAM (dimerization partner, RB-like, E2F and multi-vulval class B) complex where it represses cell-cycle dependent genes [27]. MBD4 (Methyl-CpG Binding domain protein 4) is a protein that interacts with DNMT1 and other histone modifiers and is important for repression. All four factors were more enriched at distal and intronic ARID1B bound loci than at promoter proximal binding sites, suggesting they may be involved in repression at ARID1B-regulated enhancers (S8 Fig). These subunits have not previously been tied together directly, but their co-occupancy at ARID1B alone sites suggests a potentially novel repressive complex with ARID1B.

We explored the enrichment of some factors identified in Fig 4 in more detail by comparing the binding levels of these factors at the four classes of ARID bound peaks. In most cases, peaks defined by multiple ARID binding recruited higher levels of a transcription factor or histone modification, suggesting some contribution from more than one ARID in the binding of these factors or presence of these modifications (Fig 4C). In agreement with our earlier data showing ARID1A and ARID2 were associated more with promoters, we see histone modification profiles that reflect that localization at ARID1A single and ARID2 single peaks (H3K4me3, H3K27ac, no H3K4me1). Additionally, factors predicted to cluster together at ARID1A, ARID2, and multiple bound sites, such as MAX and YY1, are present at higher levels at these peaks than at ARID1B single peaks (Fig 4C). At ARID1B single bound regions, which are more often associated with enhancers, we see high levels of H3K27ac and H3K4me1 as expected. In agreement with the clustering analysis we also find ARID1B bound peaks are enriched for higher levels of TEAD4, FOXA1, MYBL2, and HNF4*γ* compared to the ARID1A or ARID2 single bound regions (Fig 4D). Interestingly, ARID1B alone regions, but not multi regions were enriched for FOXA1 and TEAD4, suggesting these factors bind only when ARID1A or ARID2 are not present. Together, these data suggest that although binding sites for biochemically distinct SWI/SNF primarily overlap, and they share many co-factors, functional interaction with distinct transcriptional co-regulators can be revealed by examining regions bound only by a single ARID.

### De-repression of ARID1B/ARID2 regulated genes requires ARID1A

**Fig 5:**
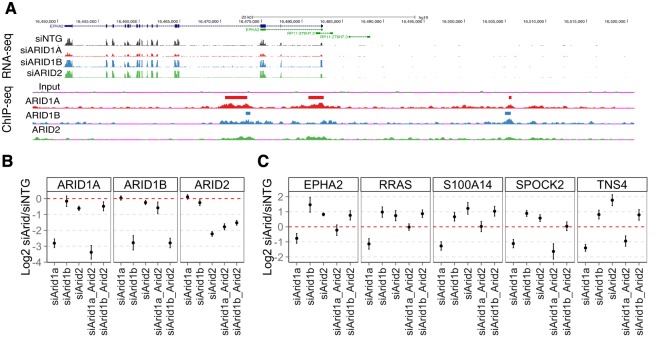
Multiple knockdown of ARID genes. A. The EPHA2 locus is an example of a gene regulated by multiple ARIDs. Colored bars depict locations of called peaks. All ChIP-seq signal intensities are normalized to reads per 30bp per 10 million mapped reads and are shown on the same relative scale. B. Single and combinatorial knockdown of ARID1A, ARID1B, and ARID2 and followed by qPCR expression measurements of the ARID genes. C. Expression changes in a panel of genes expected to be up-regulated by the loss of either ARID1B or ARID2, and down-regulated following loss of ARID1A. Error bars represent 95% confidence intervals and the experiment was repeated a minimum of 4 times.

To determine if gene expression changes following depletion of one ARID required the activity of another ARID we selected a set of genes that were significantly altered following loss of more than one of the ARID proteins and were direct targets of regulation based on ChIP-seq for multiple RNAi experiments. The most common type of functional relationship found among the ARID subunits is a repression mediated by both ARID1B and ARID2. Of 116 genes directly bound by both ARID1B and ARID2 and up-regulated following their depletion, 43 genes were down-regulated following ARID1A loss, although not to a statistically significant level in the RNA-seq data. One such example is EPHA2, where both ARID1B and ARID2 loss led to up-regulation while ARID1A loss led to down-regulation. All three ARIDs bind to sites within the promoter and in the first intron, while ARID1A and ARID1B also localize to a region upstream of the TSS (Fig 5A). We performed combinatorial knockdowns alongside singly depleted ARID subunits to determine if ARID1A and ARID2 were competitively interacting at these regions. Additionally, we performed multiple knockdown experiments targeted at ARID1B and ARID2 together to determine if they function synergistically.

We saw depletion of the single ARID subunits at a level consistent with the RNA-seq measurements, and again did not see any depletion of the non-targeted ARIDs. Knockdown of multiple ARID subunits in a single cell led to decrease in only the correct ARIDs (Fig 5B, S9A Fig). Following single knockdown of ARID1A in these five genes, we noted decreases in gene expression, and single ARID1B or ARID2 depletion caused increases in transcription levels as expected from the RNA-seq data (Fig 5C). When ARID1A and ARID2 were simultaneously depleted, expression at these genes was rescued to near wild type levels (EPHA2, RRAS, S100A14), or remained down-regulated relative to a non-targeting control (SPOCK2, TNS4) (Fig 5C). We confirmed this depedence using as second set of siRNAs (S9B and S9C Fig). This is consistent with a competitive model for ARID1A and ARID2 regulation at these genes, where ARID1A functions in transcriptional activation while ARID1B and ARID2 function in transcriptional repression.

ARID1B and ARID2 functioned concordantly nearly 100% of the time (Fig 1C), and we next looked at these concordant relationships to determine if repression by ARID1B and ARID2 were truly cooperative, or whether there was a synergistic effect of their combined depletion. Knockdown of ARID1B and ARID2 showed there was no additional de-repression from their combined loss, consistent with a cooperative relationship between ARID1B and ARID2 in the repression of these genes (Fig 5C),.

**Fig 6:**
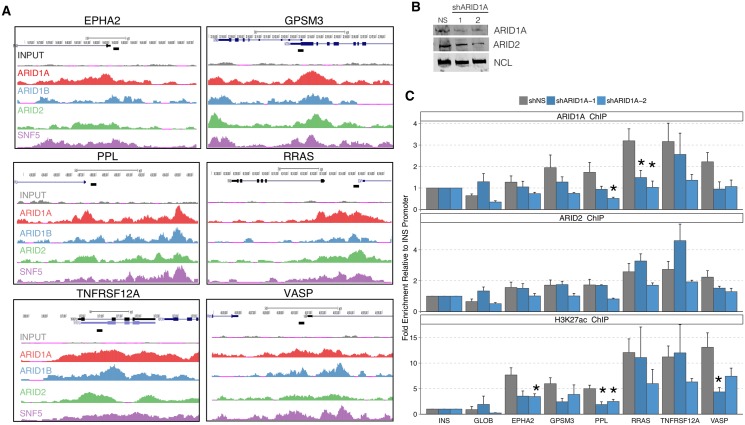
Knockdown of ARID1A leads to decrease in ARID1A binding and H3K27ac. A. Six loci were selected based on the presence of both ARID1A and ARID2 and the opposing gene expression changes associated with ARID1A and ARID2 depletion. Primer locations are noted by a black bar. B. Western blot on ARID1A and ARID2 in HepG2 cells stably transduced with a non-silencing or two ARID1A shRNAs (TRCN0000059091: shRNA-1, TRCN0000059090: shRNA-2). C. ChIP-qpcr analysis in cell lines from B. Error bars represent SEM of 3 independent experiments. Asterisk denotes p-value <0.05 by two-tailed T test.

We then tested whether loss of ARID1A from genes regulated by both ARID1A and ARID2 led to a recruitment of ARID2 and a change in the histone modifications present at these genes. We selected six genes that were bound by multiple ARIDs and were associated with decreased gene expression following ARID1A loss and increased gene expression following ARID2 loss (Fig 6A). Using two shRNAs targeting ARID1A we decreased ARID1A expression in HepG2 cells and monitored the levels of ARID1A, ARID2, and H3K27ac levels present at these genes (Fig 6B). We achieved moderate loss of ARID1A from these genes, and did not detect a consistent change in ARID2 binding at these loci (Fig 6C). This may reflect the incomplete knockdown achieved, or may be due to the relatively broad peaks of ARID associated with these genes. However, we did note a decrease in H3K27ac levels at multiple genes consistent with the requirement of ARID1A for activation at these sites (Fig 6C).

### Direct targets of ARID regulation

**Fig 7:**
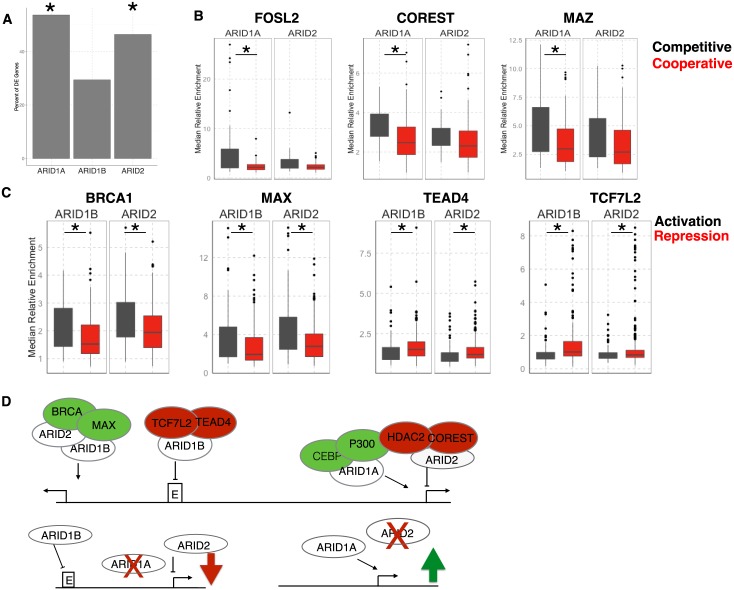
Transcription factor binding at different classes of direct ARID targets. A. Percent of differentially expressed (DE) genes associated with an ARID. Asterisk denotes statistical significance p <0.01 by hypergeometric test. B. Median signal measured at the mid-region of the peak (3kb) comparing ARID1A and ARID2 peaks associated with competitive or cooperative interactions. There were 63, 37, 58, 23 peaks associated with ARID1A cooperativity, ARID1A competition, ARID2 cooperativity, ARID2 competition respectively. C. Median signal over mid-region of peak (3kb) comparing peaks associated with activated or repressed peaks. There were 88, 287, 119, 489 peaks associated with ARID1B activation, ARID1B repression, ARID2 activation, and ARID2 repression respectively. In both B and C differences for all transcription factors and histones were compared by Wilcoxon Rank Sum Test for each set of ARID peaks comparing activated and repressed or competitive and cooperative. We adjusted p-values using Benjamini-Hochberg correction for multiple testing, asterisk indicates p-value <0.05. D. Model for combinatorial control of transcription by distinct ARID complexes.

Next we sought to determine what fraction of genes were directly regulated by ARID binding. To address this we first assigned each ChIP peak to the nearest gene and for each set of peaks we determined what fraction was associated with a gene that was altered following knockdown. This showed that 30–50% of genes were putative direct targets of the associated ARID (Fig 7A). ARID1A knockdown led to the least number of total changes in gene expression, but we found more than half (54%) of those genes were assigned to an ARID1A bound peak (p-value = 0.03, hypergeometric test) Genes misregulated following ARID1B loss were less commonly assigned as direct targets (30%, not significant), suggesting that many of the ARID1B bound regions function somewhere other than the closest gene, and consistent with a more enhancer-like localization for ARID1B. ARID2 also directly targeted a highly significant percentage of altered genes (46%, p-value = 3.4 × 10^−16^ hypergeometric test). A table of all expressed genes with transcription changes and ARID binding status can be found in BibliographyS16.

We next repeated the analysis of concordant and discordant gene expression changes (Fig 1C) to determine if the direct targets of ARID regulation also controlled similar transcriptional programs. The same pattern of both concordant and discordant regulation occurred between ARID1A and ARID1B or ARID2 (S10 Fig). Additionally, ARID1B and ARID2 direct targets shared a highly concordant pattern of transcriptional changes, consistant with the transcription changes observed for the whole gene set. The analysis of direct (S10 Fig) and potentially indirect regulatory (Fig 1) provide bounds for the ARID regulated transcriptional programs. Evaluating the ARID ChIP-seq signal shows many genes that are not associated with a peak, in fact have moderate levels of ARID binding that may reflect direct control as well (S10 Fig).

ARID1B and ARID2 alone and multiple peaks were associated with distinct genomic regions. We next asked if direct targets of ARID1B and ARID2 regulation were associated with ARID peaks at distinct genomic regions, and whether this distribution was different for activated compared to repressed genes. ARID1B peaks associated with repressed genes were more likely to be bound in an intron than were genes associated with activation following ARID1B loss, consistent with a model where ARID1B is involved in enhancer repression (S11 Fig). In contrast, there was no statistically significant difference for the ARID2 genomic localization. These data demonstrate distinctions between ARID complexes associated with activation and repression and suggest that subsetting the complex based on inclusion of a particular ARID subunit is insufficient to capture all the heterogeneity of SWI/SNF transcriptional regulation.

The co-factors associated with each of the distinct ARIDs may be unique to each type of transcriptional regulation. To address these relationships we considered two sets of directly regulated genes, those regulated by ARID1B and ARID2 and those regulated by ARID1A and ARID2. We split each set based on the relationship between the ARIDs in the RNA-seq data. For ARID1B and ARID2, we compared peaks associated with activation to those associated with repression, and for ARID1A and ARID2 we compared peaks associated with cooperation to those associated with competition. Identification of enriched transcription factors specifically associated with one of these groups relative to the others would provide evidence of heterogeneity within SWI/SNF complexes defined by inclusion of a particular ARID subunit.

Most factors bound at sites associated with ARID1A and ARID2 regulated genes were found equally at competitive and cooperative genes (S12 Fig). This is likely in part due to the low number of peaks that fit these criteria. However, we identified some differences between groups that may reflect the regulatory landscape of competitive interactions. Factors involved in transcriptional activation, such as FOSL2 (FOS-Like Antigen 2) and MAZ (MYC-Associated Zinc Finger Protein) were more enriched at sites associated with competitive interaction between ARID1A and ARID2 (Fig 7B). The higher level of these factors at the ARID1A bound regions support a role for ARID1A in transcriptional activation as observed in Fig 5C. We also noted an enrichment for the negative transcriptional regulator COREST (REST corepressor 1) at competitively regulated ARID1A and ARID2 peaks. Further, we noted enrichment at both cooperative and competitive bound peaks for other transcriptional repressors such as EZH2, HDAC2, and SIN3A (S12 Fig). The enrichment of these repressive factors is consistent with a role for ARID2 in repression of many co-regulated target genes (Fig 1B) and a model in which de-repression following ARID2 loss is dependent on ARID1A (Fig 7D).

We identified several factors that were specific for either active or repressive associated peaks (S13 Fig). For example, at genes associated with peaks of ARID1B and ARID2, that were activated by both ARID1B and ARID2, we found enrichment for BRCA1 and MAX (Fig 7C). Both have previously been found to associate with the SWI/SNF complex either directly or through genetic interaction [19, 28, 29], but have not been linked to a specific form of the SWI/SNF complex. Additionally, we found recruitment of TEAD4 associated with repression mediated by ARID1B (Fig 7C). There is a difference in TEAD4 for and TCF7L2 enrichment between ARID2 activated and repressed sites, but notably, the levels of binding associated with these sites are low, consistent with these factors being recruited specifically to ARID1B bound sites associated with transcriptional repression. These findings are consistent with a model in which ARID1B and ARID2 function coordinately to repress transcription, but may do so through binding distinct sites and using distinct co-factors. In cases where ARID1B and ARID2 coordinately activate transcription, they may function together through common binding factors (Fig 7D).

## Discussion

### Distinct SWI/SNF complexes have numerous genomic and functional interactions

Chromatin regulating proteins bind throughout the genome and play a critical role in transcriptional regulation. Studies have shown genomic co-localization for many chromatin regulators [26] as well as for different chromatin remodeling families [22]. Loss of appropriate chromatin remodeling can have genome-wide effects on transcription and chromatin structure [30]. Using three mutually exclusive subunits of the SWI/SNF complex we delineate several classes of functional relationships between biochemically distinct forms of this complex.

Using RNA-seq and ChIP-seq we classified transcriptional regulation based on the whether a gene was regulated by a single ARID containing complex, or by multiple ARID complexes. Additionally, we classified shared regulatory function based on whether two ARID proteins act concordantly or discordantly to regulate transcription. Using our ChIP-seq data combined with the rich dataset provided by the ENCODE project [18] we were able to identify differences in each of these classes.

ARID1B and ARID2 coordinately regulated transcription of a large set of genes. The majority of this overlap was seen in genes cooperatively repressed by ARID1B and ARID2. While SWI/SNF has been shown to function in repression [31–34], no study has previously shown repression mediated through a cooperative function of two distinct SWI/SNF complexes. Depletion of both ARID1B and ARID2 at the same time did not further enhance this de-repression suggesting they function in the same pathway. Many of these targets were also directly bound by ARID1B and ARID2. However, not all of these sites were occupied by both ARID1B and ARID2, which given their cooperative role in transcriptional regulation is surprising. One possibility is the cooperativity reflects a repressive role for each individually at distinct regulatory sites of a gene, and loss of a single ARID is enough to de-repress the locus. Consistent with this ARID1B peaks directly associated with repression tended to be more localized to introns, and non-overlapping with other ARIDs.

Understanding how ARID1B and ARID2 mediate these cooperative activities is an important ongoing question. Our study suggests one possible mechanism involves the co-factors recruited to each class of sites. When ARID1B and ARID2 are activating gene expression, these sites appear more commonly located near the transcription start site. Additionally, these sites are occupied by transcriptional activators such as BRCA1 and MAX, both of which have previously been linked to SWI/SNF function [19, 35], but these interactions have not been linked to a specific form of the SWI/SNF complex (Fig 7D). When ARID1B is associated with repression, it either recruits or is recruited by factors such as TEAD4 or TCF7L2 (Fig 7D). We cannot at this point determine whether the ARIDs drive recruitment of other factors to these sites or vice versa, although there is precedent for unique co-factors recruiting SWI/SNF to drive tissue specific gene activation [36]. Together this suggests that not only do ARID1B and ARID2 distinguish biochemically distinct forms of SWI/SNF (BAF-B vs PBAF complex), but the combinatorial assembly of SWI/SNF with other transcription factors and co-regulators defines functionally distinct complexes.

We identified many examples of competitive interactions amongst the ARID subunits, and validated the mechanistic importance of this competition (Fig 5C). This supports several studies that have shown transcriptional changes at single genes following loss of a SWI/SNF member depend on the remaining complex. The broad overlap we observe in transcriptional regulation (Fig 1A) suggests direct competition is a general mechanism of SWI/SNF regulatory control [4, 5, 20, 37]. Using genes that were altered by both ARID1A and ARID2 depletion, as well as were bound by both ARIDs we identified co-factors mapped by the ENCODE project that were specifically enriched near sites associated with competitive interactions compared to cooperatively regulated genes. We found both transcriptional activators, such as the MYC interacting protein MAZ, as well as a negative regulators, such as COREST at competitively regulating genes. Given the dependence on ARID1A for expression following ARID2 loss, this suggests a mechanism where ARID1A recruits or stabilizes transcriptional activators to the promoter, while ARID2 is necessary for repression (Fig 7D). Our study did not reveal a direct replacement of ARID1A by ARID2 following knockdown. However, this may be related to the difficulty of ChIP for this factors and the broad enrichment peaks. Future work evaluating swapping of biochemically distinct SWI/SNF complexes genome wide using models which allow a complete loss of one complex will shed light on the mechanisms of the different types of regulation we observe.

### Combinatorial regulation by SWI/SNF in cancer

Evidence of a central role for SWI/SNF in oncogenesis comes from the increasing variety of tumors in which the complex is recurrently mutated [14, 38–40]. In some cases loss of specific types of the SWI/SNF complex can be important in the conjunction with misregulation of certain signaling pathways. For example, a synergistic oncogenic event triggered by ARID1A loss and PIK3CA activation leads to ovarian clear cell carcinoma in an animal model [41]. Additionally, in the background of NOTCH-dependent T-ALL, PBAF complexes are required for continued tumor proliferation [42]. Both cases demonstrate an interaction between signaling pathways and chromatin regulation, however in one example, the loss of a SWI/SNF complex is necessary for tumor formation, while in the second a specific SWI/SNF complex is required for continued tumor growth. Finally, some forms of the SWI/SNF complex have been shown to have direct oncogenic effects through their role in transcriptional regulation. In synovial sarcoma a fusion of a SWI/SNF subunit (SS18) to SSX leads to the eviction of SNF5 from the complex and mis-localization of SWI/SNF ultimately resulting in inappropriate activation of a proliferative transcriptional program [43]. These recent mechanistic insights into SWI/SNF mediated mis-regulation in cancer highlight the importance of understanding the functional relationships between the many possible forms of this complex.

The idea of therapeutically targeting SWI/SNF, either in the context of other SWI/SNF mutations, or in tumors with specific genetic lesions, is beginning to gain traction [17, 19]. When considering how these synthetic lethalities manifest, it is important to consider how the remaining subunits of SWI/SNF contribute to the overall transcriptional program of a cell. We identify widespread functional interaction between distinct forms of the SWI/SNF complex that in many cases depends on the remaining SWI/SNF in the cell. Given the broad role of SWI/SNF in gene regulation (21% of genes expressed in HepG2 cells), it is likely that these types of interactions are common to other cell types, and the discovery of more interactions will shed light on the functional relationships within the SWI/SNF complex. The existence of both competitive and cooperative interactions between distinct SWI/SNF complexes provides mechanistic clues into synthetic lethality involving SWI/SNF and is an intriguing opportunity to identify new therapeutic targets specific to mis-regulation of the complex [17, 19].

## Materials and Methods

### Cell culture

HepG2 cells (UNC Tissue Culture Facility) were grown in DMEM (Gibco, Life Technologies) supplemented with 10% Fetal Calf Serum and penicillin/streptomycin (100units/mL, Life Technologies).

### Antibodies

Arid1a: Bethyl A301–041A (western), Santa Cruz sc-32761x (ChIP). Arid1b: Abcam ab57461 (western), Santa Cruz sc-32762x (ChIP). Arid2 Bethyl A302–230A (western/ChIP). Nucleolin Bethyl (A300–711A). H3K27ac (ChIP) Abcam ab4729.

### siRNA and shRNA knockdown experiments

*siRNA* 2 × 10^6^ HepG2 cells were grown as above and reverse transfected with RNAimax reagent (Life Technologies) using 30nM siRNA and 10uL RNAimax in 6cm dishes at 6.6 × 10^5^ /mL. After 72 hours of incubation RNA was harvested using Trizol (Life Technologies) and further purified using a RNAeasy clean up kit (Qiagen). siRNAs used were—Arid1a (siGenome L-017263-00-0005 (Dharmacon), Silencer Select s15786 (Life Technologies), Arid1b (siGenome M-013970-00-0005 (Dharmacon), 1027416 (Qiagen)), Arid2 (Silencer Select s47042(Life Technologies), siGenome M-026945-01-0005 (Dharmacon), Non-targeting Control (AM4611, Life Technologies).

*shRNA* shRNA constructs targeting ARID1A (TRCN0000059091: shRNA-1, TRCN0000059090: shRNA-2) and a non-silencing construct were used to generate lentivirus by transfecting HEK293T cells with the pLKO.1 lentiviral constructs, pMD2.G and psPAX2 (psPAX2 and pMD2.6 were a gift from Didier Trono—Addgene plasmid # 12260 and plasmid # 12259). After 72 hours viral supernatent was harvested concentrated using Lenti-X (Clontech). Knockdowns for ChIP experiments were generated by transducing HepG2 cells on 6 cm plates and selecting for puromycin resistance starting 48 hours post-transduction. Cells for ChIP were handled as described below.

### Chromatin immunoprecipitation

Prior to starting ChIP, 5–10ug antibody were incubated with 50uL Dynal beads (Life Technologies), for rabbit antibodies we used a 1:1 mix of protein A and protein G beads, for mouse antibodies protein G only. Antibody/bead mixture was incubated overnight at 4°C in 0.5% Bovine Serum Albumin (BSA) in PBS. 50–100 × 10^6^ cells were fixed in 0.3% formaldehyde for 30 minutes at 4°C and then quenched with 0.125M glycine for 5 minutes at room temperature. Pellets were washed three times in cold PBS and frozen in liquid nitrogen and stored at -80°C. For each ChIP 2 × 10^7^ cells were thawed on ice and resuspended in 1mL swelling buffer (25mM Hepes, 1.5mM MgCl_2_, 10mM KCl, 0.1% NP-40) with 1mM PMSF and incubated 10 minutes on ice. Pellets were dounced 20 strokes with B pestle and spun at 2000RPM for 7 minutes at 4°C in a swinging bucket centrifuge. Cell pellets then resuspended in 5mL sucrose buffer A (0.32M Sucrose, 15mM Hepes pH 7.9, 50mM KCl, 2mM EDTA, 0.5mM EGTA, 0.5mM PMSF) and layered gently over 5mL sucrose buffer B (30% Sucrose, 15mM Hepes pH 7.9, 50mM KCl, 2mM EDTA, 0.5mM EGTA, 0.5mM PMSF) and centrifuged 10 minutes at 3000 RPM in a swinging buffer centrifuge at 4°C. Nuclei were washed once in 10mL digestion buffer (15mM Hepes ph7.9, 60mM KCl, 15mM NaCl, 0.32M Sucrose, 0.5mM PMSF) and pelleted for 10 minutes at 2000 RPM. They were then resuspended in 1mL per 4 × 10^7^ cells with 3.3uL CaCl_2_ per mL with 1X protease inhibitor (Roche) and 1mM PMSF and incubated for 5 minutes at 37°C. 0.5uL MNAse (NEB, 2 × 10^6^ units/mL) was added to tubes and incubated 15 minutes at 37°*C*. MNase digestion was stopped by adding 0.1 volumes 0.5M EDTA and reactions were incubated for 5 minutes on ice. Nuclei were then passed 5 times through a 20G needle, 1 volume of double concentration LB3 (20mM Tris-HCl pH 8.0, 100mM NaCl, 1mM EDTA, 0.5mM EGTA, 0.1% Na-Deoxycholate, 0.5% N-lauroylsarcosine) was added and lyaste was passed 5X through 25G needle. Triton X-100 was added to final concentration of 1%, and cells were spun at 13000G in microfuge at 4° for 15 minutes. Supernatant was saved, and the pellet was resuspended in 1X LB3 buffer with 1% Triton X-100 and incubated 1–2 hours rotating at 4°. Following another 13000G spin, the supernatants were combined and 10% was reserved as input DNA. 1mL (equivalent to 20 × 10^6^ cells) was added to the prepared beads and incubated overnight. Beads were washed 8 times in RIPA (50mM Hepes-KOH, pH 7.6, 500mM LiCl, 1mM EDTA, 1% NP-40, 0.7% Na-deoxycholate) and 1 time in TE (10mM Tris-HCl pH 8.0, 1mM EDTA) supplemented with 50mM NaCl before elution in 100uL 1%SDS, 100mM NaHCO_3_ for 17 minutes at 65 degrees with agitation. Supernatant was removed and supplemented with 5uL of 5M NaCl and incubated overnight at 65°C. 3uL RNAseA(30mg/mL) was added to reactions and incubated for 30 minutes at 37°C, followed by 5ul Proteinase K (20mg/mL) at 56° for 1 hour. Reactions were purified using a Zymo Clean and Concentrator Chip Kit (D5205) and concentration was measured using qubit (Life Technologies) before library preparation.

### Co-immunoprecipitation

Small-scale nuclear extracts were prepared as previously described [20]. Immunoprecipitations were performed by incubating antibodies to ARID1A, ARID1B, ARI2, or IgG overnight at 4° followed by incubated for 2 hours with M280 Sheep-anti rabbit more mouse beads (Life Technologies). The beads were washed 4 times with 150mM Nacl, twice with 100mM NaCl, and once with 60mM NaCl before eluting in 1X laemmli loading buffer by boiling for 10 minutes.

### RNA-seq library prep

RNA-seq library preparation was done using the dUTP incorporation method modified from [44]. Briefly, 30ug total RNA was poly A+ selected using Dynal poly A+ beads. RNA was then fragmented using Ambion Fragmentation Reagent (AM8740, Life Technologies) for 4 minutes at 70 degrees and cleaned using a Zymo ssDNA/RNA kit (D7010). The fragmented RNA was used in a first strand synthesis reaction using NEB random primers (S1330S, NEB) and Superscript III (18080044, Life Technologies) following manufacturers instructions. Reaction was then purified and used in a second strand synthesis reaction with dUTP replacing dTTP in the nucleotide mix for 2 hours at 16°C. The reaction was purified with 1.8 volumes Ampure XP beads (A63880, Beckman-Coulter) and used in standard Illumina Library Preparation (See section below), except prior to final amplification libraries were treated with Uracil DNA-Glycosylase (M0280S, NEB) to degrade the d-UTP marked second strand.

### Library prep

We prepared libraries using above cDNA or ChIP DNA for Illumina sequencing using standard protocols and NEB Next Reagents. Briefly, DNA ends were repaired using end repair module (E6050S, NEB). Then molecules were A-Tailed using Klenow exo^−^ and Illumina Adapters were ligated to molecules using the Quick Ligation Module (E6056S). Libraries were then purified twice with Ampure XP beads (1 volume first round, 0.8 volumes second round. Libraries were then amplified using NEBNExt Q5 polymerase and quantitated and submitted for Illumina Sequencing at the University of North Carolina at Chapel Hill High Throughput Sequencing Facility.

### Data analysis

Data were processed using R 3.0 [45] and Python 2.7.6 (http://python.org) and packages indicated below. Code used in analysis is available at http://github.com/jraab/raab_swisnf_2015.

### ChIP-seq analysis

Single end 50bp reads from two independent experiments were aligned to hg19 using bowtie2 (2.2.1) (http://bowtie-bio.sourceforge.net/bowtie2/index.shtml [46] filtered for duplicates and merged using samtools (0.1.19) [47] prior to final peak calling. Peaks were called using MACS2 (https://github.com/taoliu/MACS/ [48]) using –broad -q 0.01 settings. Peaks were further processed by using pybedtools [49, 50] to merge all peaks within 100 basepairs of each other and removing any peaks that are found in the ENCODE blacklist regions. Each peak was assigned to the nearest gene and the relationship to genomic features was assigned using Gencode V16 for gene annotations.

### RNA-seq analysis

Single-end 50bp reads were aligned to hg19 using Tophat2 (2.0.13) [51] and htseq-count using -s reverse -m intersection-strict -i gene_name (0.5.4p5) [52] was used to process aligned reads into gene level counts using Gencode V16 annotations. Differential expression was tested usinge DESeq2 (1.6.3) [53]. For UCSC Genome Browser Tracks replicates were merged and converted to strand specific bigwig files.

### Chromatin module identification

Data sets for transcription factors and histone modifications were obtained from the ENCODE portal (http://genome.ucsc.edu/ENCODE/downloads.html) and were downloaded as aligned reads (BAM format). Replicates were merged and signal centered on each ARID peak was calculated using python scripts (available in github repository). Median signal over each peak was used to calculate a pairwise correlation (Spearman) for each pair of ChIP-seq peaks. These were clustered (1-cor) and visualized using the gplots::heatmap.2 function in R. Clusters were assigned using k-means (k = 9) and the network diagram of similarities between clusters was derived from the number of shared transcription factors (edges) between any two clusters (nodes). We removed any clusters with a median enrichment of <0.1.

### RT-PCR

RT-PCR was performed by reverse transcribing RNA using Multiscribe (Life Technologies—4311235), followed by quantitative PCR using Ssofast EvaGreen Supermix (Bio-Rad—172-5203) reagents per manufacturers instructions on a Bio-Rad CFX-96 machine. Primer sequences were as follows: Arid1a-F AGATGGGACACCCAAGACAG, Arid1a-R CTTCCTCTCAGGCTCACCAC; Arid1b-F TCTCCTGTTGGCTCTCCTGT, Arid1b-R GTGTGCCTGCCATAAAACCT; Arid2-F GGAGCAGATCCAAGCACTGT Arid2-R GACTGCCACAGACACATGAAG; Epha2-F AGGCTACGAGAAGGTGGAGG, Epha2-R CAAGCAGGGGCTCTCAGAT; S100A14-F TGGCTCCTCCTGTCTTGTCT S100A14-R CCCACTGTGTCTGGTCCTTT; Spock2-F TGCCAGAAGGTGAAGTGCAG Spock2-R TTTCCATGGAGTTTCACGGT; TNS4-F AGCTCTTCATACCGAGGCTC, TNS4-R CTTTGGCAGACGACTCGATG. AVIL-F AGGCAGAGCACTACCTGTGG; AVIL-R AGTGTTCTCGCTGCCATCA; AXL-F CCGTAACCTCCACCTGGTC; AXL-R ATCCCATCGTTTGACAGCAC; CD109-F TTTCCTCCTAATACAGTGACTGGC; CD109-R CCATTGATGGAAGGACCAAG; EPHA1-F GGAGAGTGACCAGGATGTGG; EPHA1-R ACACAAGGTCTCGAATGGTGA; S100A3-F AAATACAAGCTCTGCCAGGC; S100A3-R GTAGAGACAGAGGCAGGCAA; FOXM1-F AGGAGAATTGTCACCTGGAGC; FOXM1-R GTAAGAGTAGGGTGGCCGCT; KISS1R-F AACTCGCTGGTCATCTACGT; KISS1R-R AACTTGCACATGAAGTCGCC; MCM7-F GCGATGGCACTGAAGGACTA; MCM7-R TTCCCGAGTTCATCATCCTG; TIE1-F GACTGACCCAGCTTTTGCTC; TIE1-R CTGCAATCTTGGAGGCTAGG; All experiments were normalized to expression of Gusb (Gusb-F AAACGATTGCAGGGTTTCAC, Gusb-R CTCTCGTCGGTGACTGTTCA), and presented as the log2 ratio of siArid/siNTG.

### Pathway analysis

Gene lists were compared to the MSigDB database (downloaded—2015-05-05). P-values were generated using the hypergeometric test, using all possible genes in the MSigDB as the background and were adjusted using the Benjanmi-Hochberg correction for multiple testing.

### Ethics statement

All animal work was conducted according to relevant national and international guidelines and was approved by UNC Chapel Hill’s IACUC

### Accession numbers

Raw and processed data can be found at GEO GSE69568—Analysis scripts can be found at http://github.com/jraab/raab_swisnf_2015 All sequencing Data are also available as a UCSC Genome Browser Trackhub using http://genome.ucsc.edu/cgi-bin/hgTracks?db=hg19&hubUrl=http://trackhubs.its.unc.edu/magnuslb/jraab/swi_snf/hub.txt

## Supporting Information

S1 Fig Relative Expression of ARID subunits in cancer cell lines. ARID1A, ARID1B, and ARID2 TPM values from ENCODE experiments [18]. The red line indicates the average of the ENCODE experiments for that cell type.
(EPS)

S2 Fig Efficiency of ARID knockdown. A. Western blot of protein levels of each ARID following knockdown. B. Expression values (log2 Fold Change) measured by RNA-seq for each ARID following loss of each ARID.
(EPS)

S3 Fig Validation of RNA-seq by qPCR. A-C. Validation of RNA-seq data using the primary set of siRNA used in RNA-seq (siRNA 1) and a second siRNA set (siRNA 2) at selected genes following knockdown of ARID1A (A), ARID1B (B), and ARID2 (C). Error bars represent standard error of the mean for 2 independent experiments.
(EPS)

S4 Fig Overlap of ChIP-seq peaks. A-C. Overlap of ARID1A, ARID1B, or ARID2 with SNF5/BAF47, demonstrating fraction of ARID peaks associated with core SWI/SNF factor by peak calls. D. Union of all ARID peaks overlapping SNF5/BAF47 peaks. E. Comparison of SNF5/BAF47 peak calls in our manuscript with those in Euskirchen et. al. [23].
(EPS)

S5 Fig Histone modifications associated with ARID bound regions. H3K4me1, H3K4me3, and H3K27ac signal was measured centered on the ARID bound regions using information from the ENCODE project [18, 55]. Data are represented as the average signal which light shading depicting the 95% confidence interval.
(EPS)

S6 Fig Comparison of signal between different categories of ARID peaks. The signal at each set of ARID peaks (ARID1A alone—top panels, ARID1B alone—second panels, ARID2 alone—third panels, Multiple peaks—bottom panels), for each of the ARIDs and SNF5/BAF47 (ARID1A—red, ARID1B- blue, ARID2- green, SNF5- purple) +/- 2kb from the midpoint of the peak to demonstrate the accuracy of the ‘Alone’ vs ‘Multiple’ peak definitions.
(EPS)

S7 Fig Correlation analysis of transcription factors and histone modifications at ARID bound regions. The average enrichment for each of 77 ENCODE mapped factors [18] at the four classes of ARID peaks (ARID1A alone (A), ARID1B alone (B), ARID2 alone (C), and multiple bound (D); green track on left is enrichment). For each of these factors we compared the enrichment at each set of arid peaks using Spearman Correlation coefficients and clustered these data to yield the middle heatmaps showing which sets of signals were most related. We used k-means clustering (k = 9) to define interesting clusters. Red is highly correlated, Blue is anti correlated. Important clusters from main Fig 4A are labelled.
(EPS)

S8 Fig Genomic location of factors found in Arid1b-specific cluster. We calculated the signal enrichment for each of the members of the ARID1B-specific cluster (MYBL2, MBD4, TEAD4, and NFIC) at three types of genomic features (exons were excluded because too few sites existed, which led to very high noise in that data). The top row consists of ARID1B-multiple ARID sites, and the bottom row is ARID1B-single ARID sites. The colors distinguish the 3 genomic locations of these peaks. Solid line represents average signal intensity with the shaded region denoting the 95% confidence interval centered at the midpoint of the ARID1B peak +/- 2.5kb.
(EPS)

S9 Fig Validation of ARID1A dependence on gene expression changes following ARID2 loss. A. Western blot of combinatorial knockdown using siRNA set #1 from Fig 5B. B. qPCR validation of single and combinatorial knockdown of ARID1A and ARID2 using siRNA set #2. C. qPCR validation of gene expression changes and ARID1A dependence for gene activation at competitively regulated genes following combinatorial knockdown of ARID1A and ARID2 using siRNA set #2. Error bars represent standard error of the mean, n = 2.
(EPS)

S10 Fig Pairwise gene expression changes of directly bound ARID targets. A. Pairwise gene expression changes of ARID1A and ARID1B directly bound co-regulated genes. B. ARID1A ChIP-seq enrichment at all genes significantly regulated by ARID1A and ARID1B C. ARID1B ChIP-seq enrichment at all genes significantly regulated by ARID1A and ARID1B D. Pairwise gene expression changes of ARID1A and ARID2 directly bound co-regulated genes. E. ARID1A ChIP-seq enrichment at all genes significantly regulated by ARID1A and ARID2. F. ARID2 ChIP-seq enrichment at all genes significantly regulated by ARID1A and ARID2 G. Pairwise gene expression changes of ARID1B and ARID2 directly bound co-regulated genes. H. ARID1B ChIP-seq signal at all genes significantly regulated by ARID1B and ARID2. I. ARID2 ChIP-seq signal at all genes significantly regulated by ARID1B and ARID2.
(EPS)

S11 Fig Genomic location of peaks associated with ARID1B and ARID2 activation or repression. Percent of ARID1B or ARID2 peaks that are involved in activation or repression that localize to different classes of genomic features. The shift towards distal/intronic peaks in the repressed category is statistically significant for ARID1B peaks(Chi Squared p-value = 0.02), but not for ARID2 peaks (p-value = 0.08). N = 72, 233, 80, 314 for ARID1B activated, ARID1B repressed, ARID2 activated, ARID2 repressed, respectively.
(EPS)

S12 Fig Enrichment of transcription factors and histone modifications at peaks associated with cooperation or competition. These data underly the selection of genes in Fig 7B. We compared the mean signal values for different histone modifications and transcription factors at ARID1A or ARID2 peaks associated with genes we classified as competitively or cooperatively regulated. We used these data to identify transcription factors or histone modifications specifically associated with a particular mode of regulation.
(EPS)

S13 Fig Enrichment of transcription factors and histone modifications at peaks associated with activation or repression. These data underly the selection of genes in Fig 7C. We compared the mean signal values for histone modifications and transcription factors at ARID1B and ARID2 peaks associated with either activation or repression. These data were used to identify factors that were more enriched when associated with repressed compared to active regions or vice versa.
(EPS)

S1 Table Additional file 1—ARID1A bound regions. Bed file of ARID1A bound regions (hg19).
(BED)

S2 Table Additional file 2—ARID1B bound regions. Bed file of ARID1B bound regions (hg19).
(BED)

S3 Table Additional file 3—ARID2 bound regions. Bed file of ARID2 bound regions (hg19).
(BED)

S4 Table Additional file 4—SNF5 bound regions. Bed file of SNF5 bound regions (hg19).
(BED)

S5 Table Additional file 5—ENCODE data sets used. (CSV)

S6 Table Additional file 6—All expressed genes with associated ARID transcription changes and peak information. CSV file of all expressed genes and the transcriptional changes associated with each ARID at that gene and whether an ARID peak is assigned to that gene.
(CSV)

S7 Table Additional file 7—Pathway analysis of ARID1A regulated genes. Excel file with pathway analysis of alone and jointly regulated ARID1A genes.
(XLSX)

S8 Table Additional file 8—Pathway analysis of ARID1B regulated genes. Excel file with pathway analysis of alone and jointly regulated ARID1B genes
(XLSX)

S9 Table Additional file 9—Pathway analysis of ARID2 regulated genes. Excel file with pathway analysis of alone and jointly regulated ARID2 genes.
(XLSX)

S10 Table Additional file 10—All ARIDs differentially expressed genes. CSV file containing all significantly changed genes following ARID knockdown. Contains ‘Alone’ or ‘Jointly’ regulated label.
(CSV)

S1 Text Supplementary figures and text. Single PDF of additional figures and text.
(PDF)
