# Supplementary material for: Genome-Wide Transcriptional Regulation Mediated by Biochemically Distinct SWI/SNF Complexes
Source: PLoS Genet. 2015 Dec 30;11(12):e1005748. doi: 10.1371/journal.pgen.1005748 (PMC4699898; doi:10.1371/journal.pgen.1005748)
Supplement: S1 Text — Single PDF of additional figures and text. (PDF) [file pgen.1005748.s024.pdf]

Supplemental Figure 1

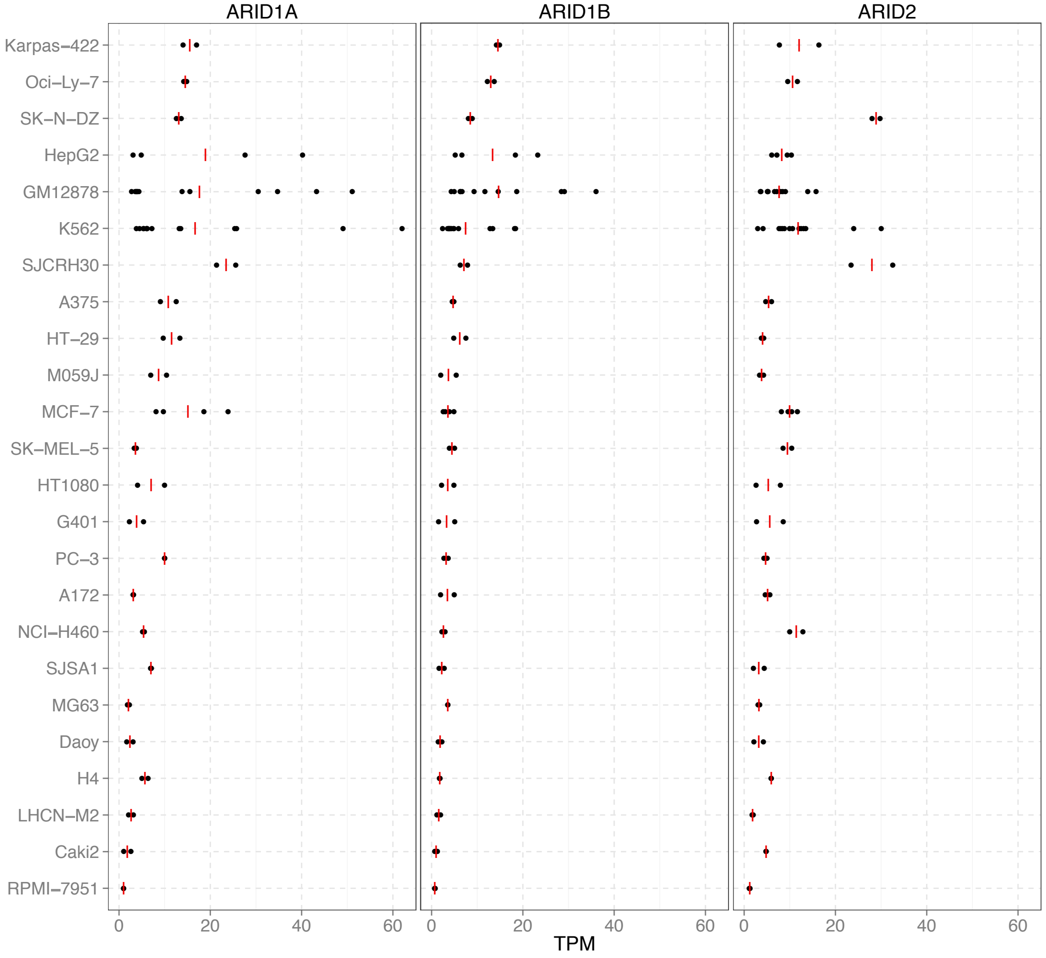

Supplemental Fig. 1: Relative Expression of ARID subunits in cancer cell lines ARID1A, ARID1B, and ARID2 TPM values from ENCODE experiments [1]. The red line indicates the average of the ENCODE experiments for that cell type.

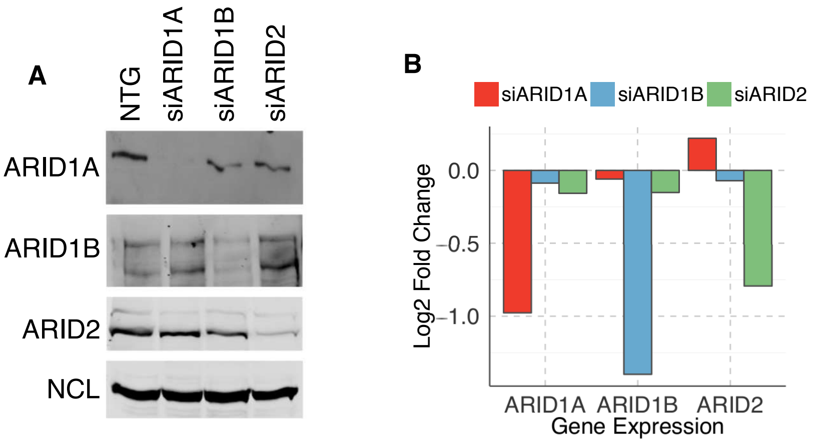

Supplemental Fig. 2: Efficiency of ARID knockdown
A. Western blot of protein levels of each ARID following knockdown. B. Expression values (log2 Fold Change) measured by RNA-seq for each ARID following loss of each ARID.

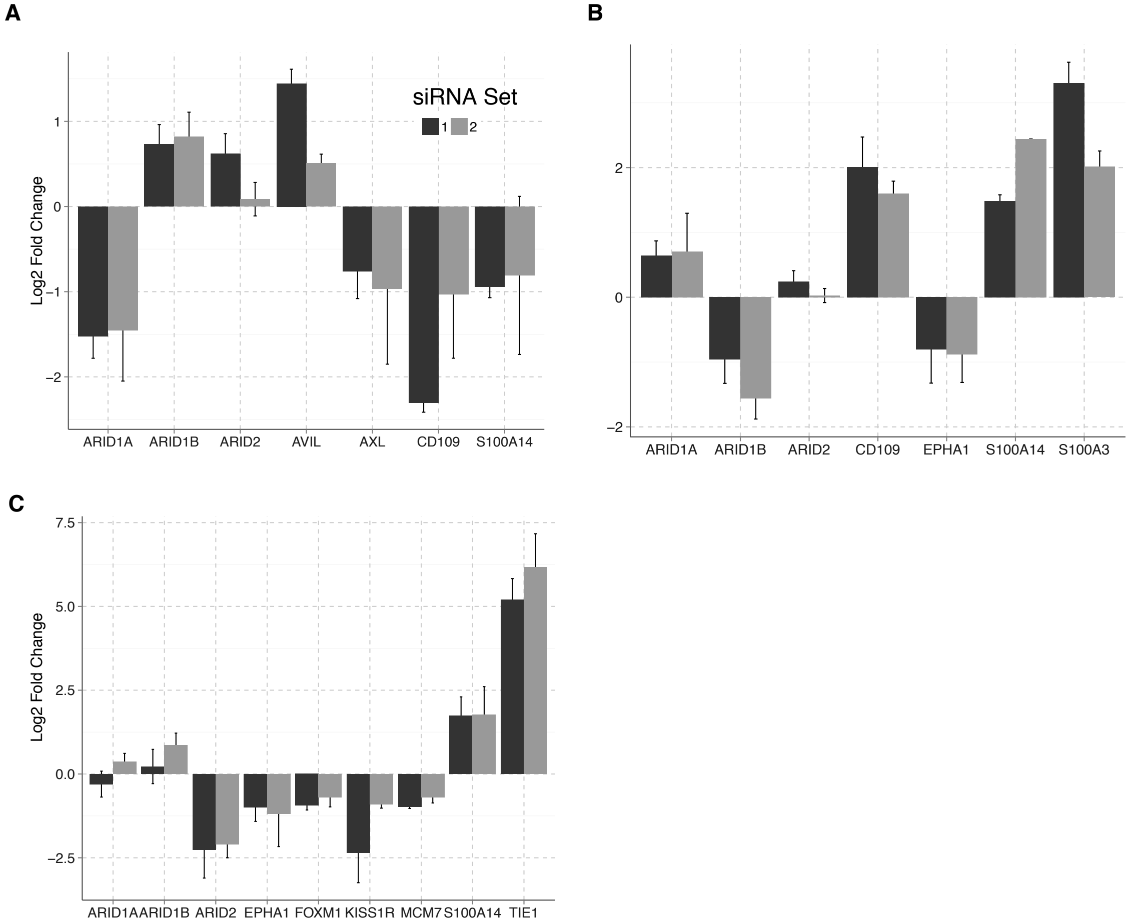

Suppelmental Fig. 3: Validation of RNA-seq by qPCR A-C. Validation of RNA-seq data using the primary set of siRNA used in RNA-seq (siRNA 1) and a second siRNA set (siRNA 2) at selected genes following knockdown of ARID1A (A), ARID1B (B), and ARID2 (C). Error bars represent standard error of the mean for 2 independent experiments.

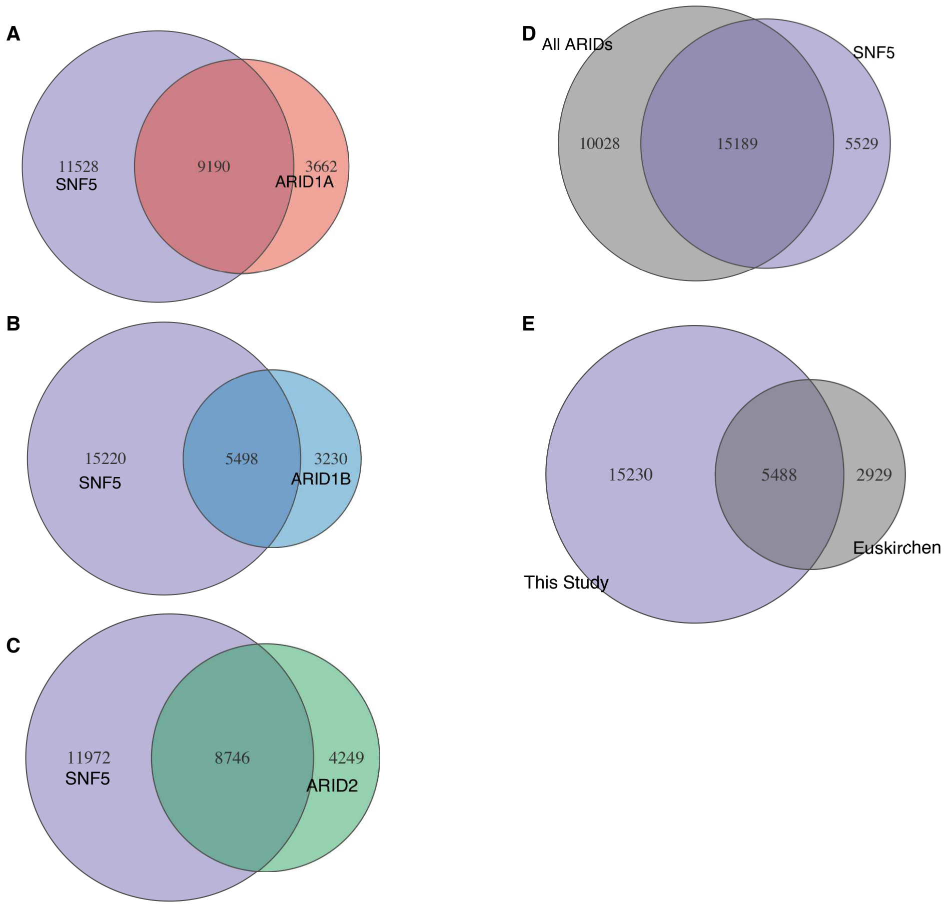

Supplemental Fig. 4: Overlap of ChIP-seq peaks
A-C. Overlap of ARID1A, ARID1B, or ARID2 with SNF5/BAF47, demonstrating fraction of ARID peaks associated with core SWI/SNF factor by peak calls. D. Union of all ARID peaks overlapping SNF5/BAF47 peaks. E. Comparison of SNF5/BAF47 peak calls in our manuscript with those in Euskirchen et. al. [2].

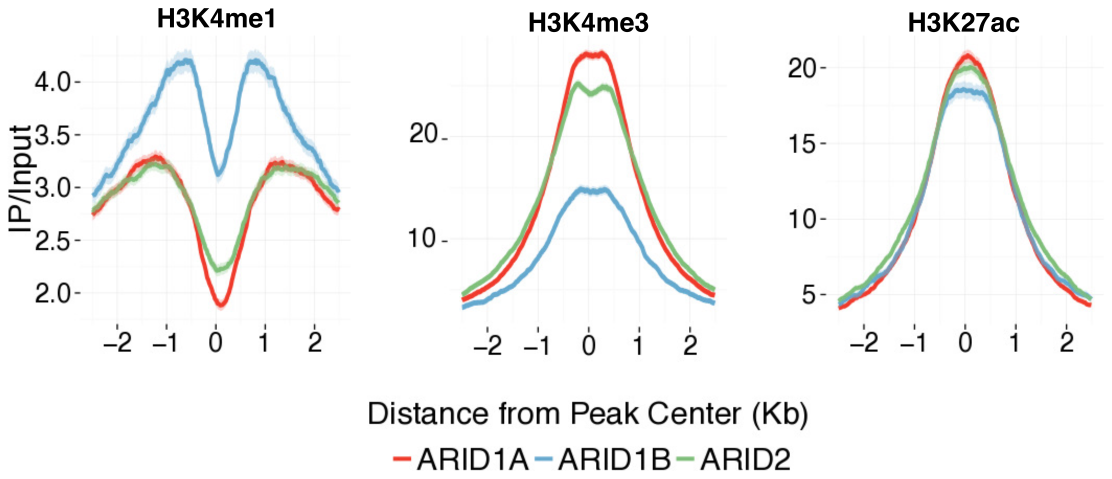

Supplemental Fig. 5: Histone modifications associated with ARID bound regions
H3K4me1, H3K4me3, and H3K27ac signal was measured centered on the ARID bound regions using information from the ENCODE project [3, 1]. Data are represented as the average signal which light shading depicting the 95% confidence interval.

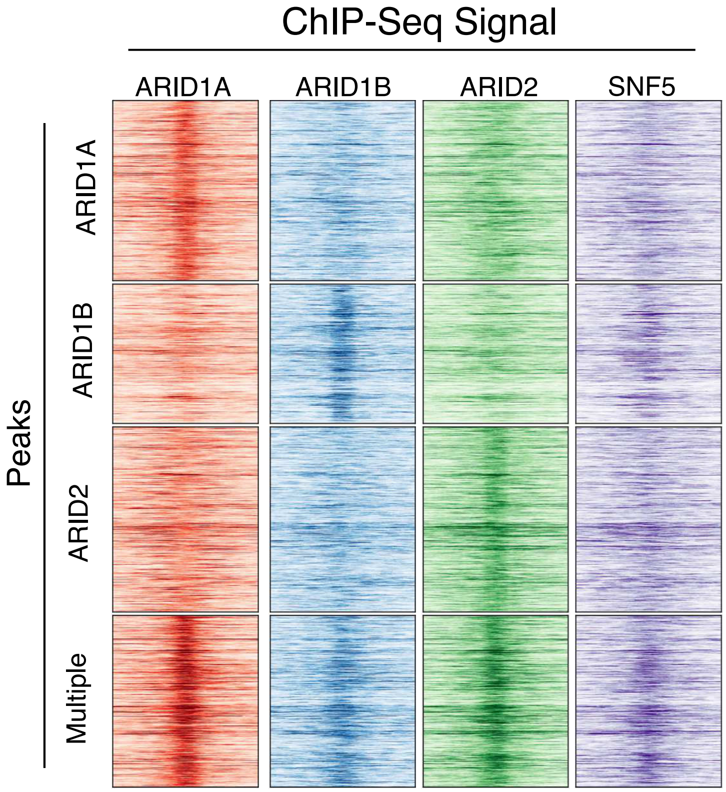

Supplemental Fig. 6: Comparison of signal between different categories of ARID peaks
The signal at each set of ARID peaks (ARID1A alone - top panels, ARID1B alone - second panels, ARID2 alone - third panels, Multiple peaks - bottom panels), for each of the ARIDs and SNF5/BAF47 (ARID1A - red, ARID1B- blue, ARID2- green, SNF5- purple) +/- 2kb from the midpoint of the peak to demonstrate the accuracy of the 'Alone' vs 'Multiple' peak definitions.

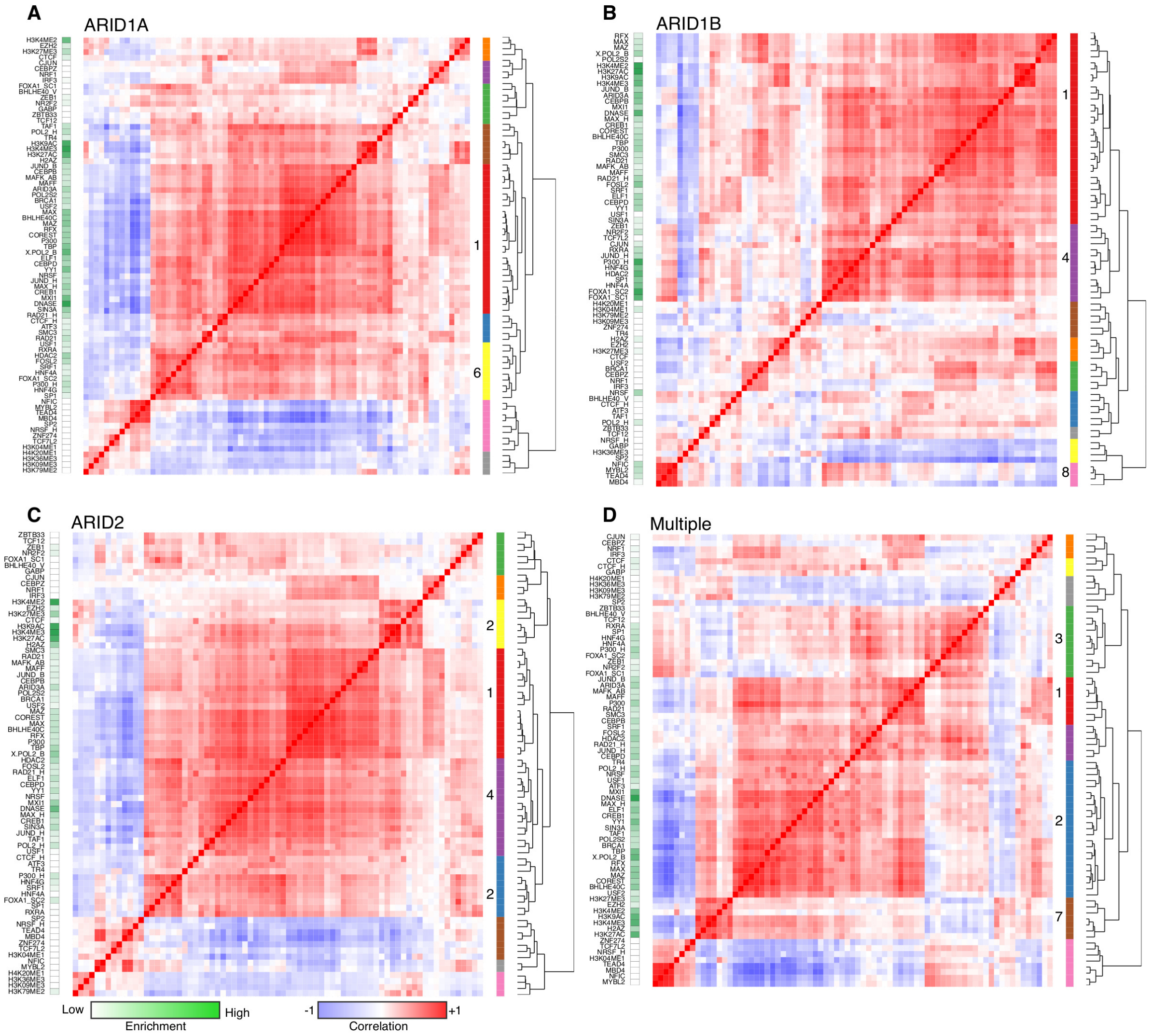

Supplemental Fig. 7: Correlation analysis of transcription factors and histone modifications at ARID bound regions

The average enrichment for each of 77 ENCODE mapped factors [1] at the four classes of ARID peaks (ARID1A alone (A), ARID1B alone (B), ARID2 alone (C), and multiple bound (D); green track on left is enrichment). For each of these factors we compared the enrichment at each set of arid peaks using Spearman Correlation coefficients and clustered these data to yield the middle heatmaps showing which sets of signals were most related. We used k-means clustering (k=9) to define interesting clusters. Red is highly correlated, Blue is anti correlated. Important clusters from main Fig. 4A are labelled.

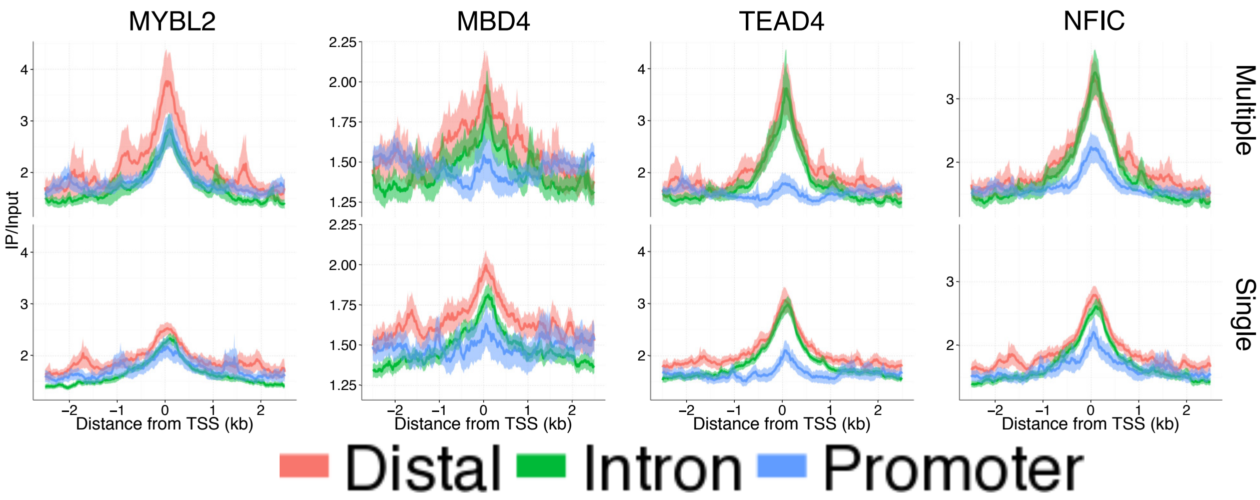

Supplemental Fig. 8: Genomic location of factors found in Arid1b-specific cluster
We calculated the signal enrichment for each of the members of the ARID1B-specific cluster (MYBL2, MBD4, TEAD4, and NFIC) at three types of genomic features (exons were excluded because too few sites existed, which led to very high noise in that data). The top row consists of ARID1B-multiple ARID sites, and the bottom row is ARID1B-single ARID sites. The colors distinguish the 3 genomic locations of these peaks. Solid line represents average signal intensity with the shaded region denoting the 95% confidence interval centered at the midpoint of the ARID1B peak +/- 2.5kb.

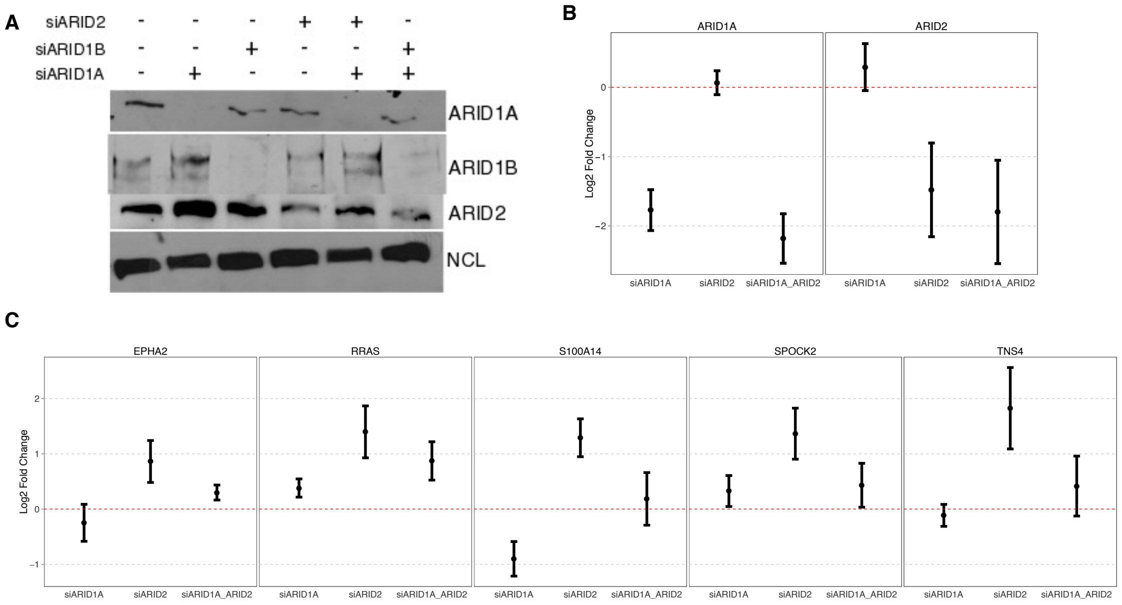

Supplemental Fig. 9: Validation of ARID1A dependence on gene expression changes following ARID2 loss
A. Western blot of combinatorial knockdown using siRNA set 1 from Figure 5B. B. qPCR validation of single and combinatorial knockdown of ARID1A and ARID2 using siRNA set 2. C. qPCR validation of gene expression changes and ARID1A dependence for gene activation at competitively regulated genes following combinatorial knockdown of ARID1A and ARID2 using siRNA set 2. Error bars represent standard error of the mean, n = 2.

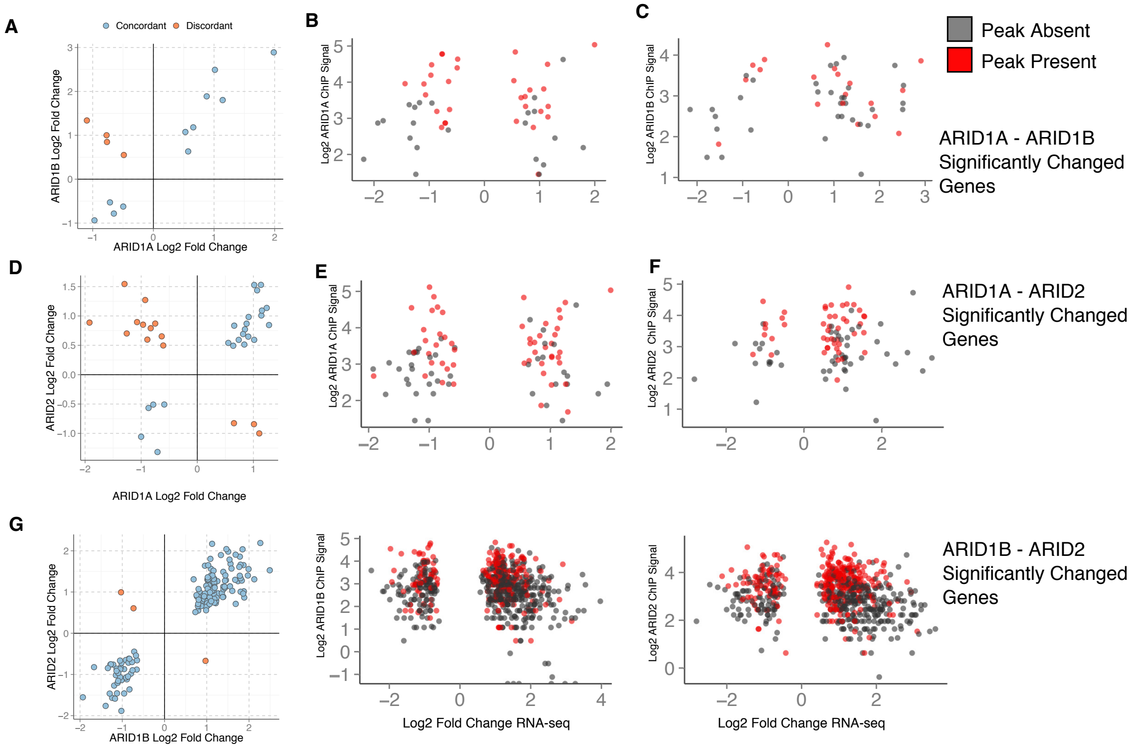

Supplemental Fig. 10: Pairwise gene expression changes of directly bound ARID targets A. Pairwise gene expression changes of ARID1A and ARID1B directly bound co-regulated genes. B. ARID1A ChIP-seq enrichment at all genes significantly regulated by ARID1A and ARID1B C. ARID1B ChIP-seq enrichment at all genes significantly regulated by ARID1A and ARID1B D. Pairwise gene expression changes of ARID1A and ARID2 directly bound co-regulated genes. E. ARID1A ChIP-seq enrichment at all genes significantly regulated by ARID1A and ARID2. F. ARID2 ChIP-seq enrichment at all genes significantly regulated by ARID1A and ARID2 G. Pairwise gene expression changes of ARID1B and ARID2 directly bound co-regulated genes. H. ARID1B ChIP-seq signal at all genes significantly regulated by ARID1B and ARID2. I. ARID2 ChIP-seq signal at all genes significantly regulated by ARID1B and ARID2.

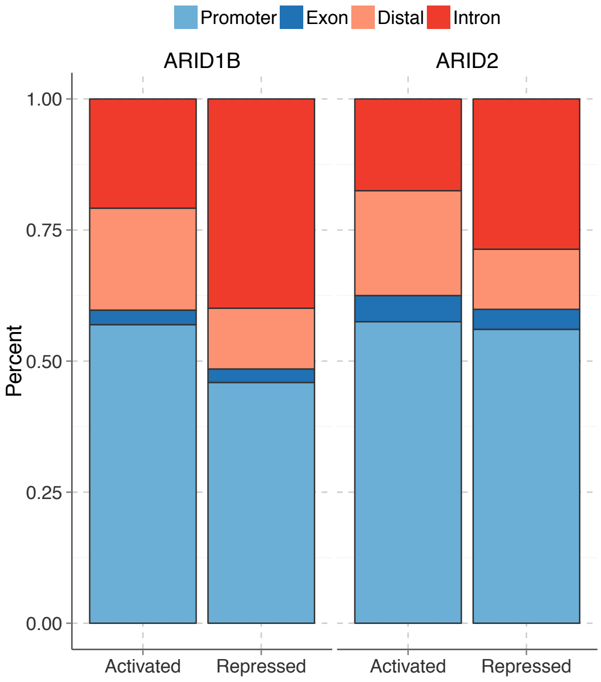

Supplemental Fig. 11: Genomic location of peaks associated with ARID1B and ARID2 activation or repression

Percent of ARID1B or ARID2 peaks that are involved in activation or repression that localize to different classes of genomic features. The shift towards distal/intronic peaks in the repressed category is statistically significant for ARID1B peaks(Chi Squared p-value = 0.02), but not for ARID2 peaks (p-value = 0.08). N = 72, 233, 80, 314 for ARID1B activated, ARID1B repressed, ARID2 activated, ARID2 repressed, respectively.

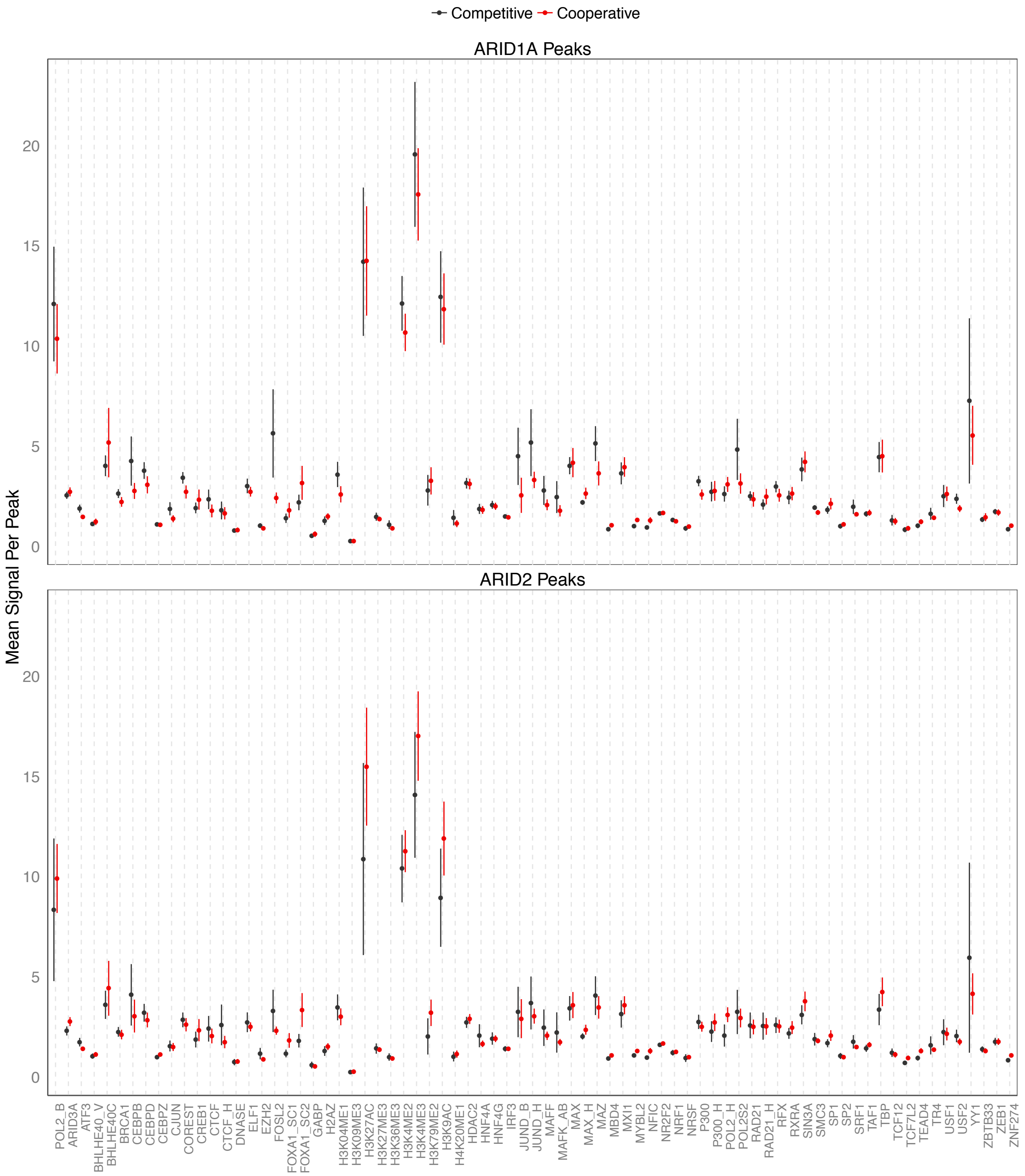

Supplemental Fig. 12: Enrichment of transcription factors and histone modifications at peaks associated with cooperation or competition

These data underly the selection of genes in Figure 7B. We compared the mean signal values for different histone modifications and transcription factors at ARID1A or ARID2 peaks associated with genes we classified as competitively or cooperatively regulated. We used these data to identify transcription factors or histone modifications specifically associated with a particular mode of regulation.

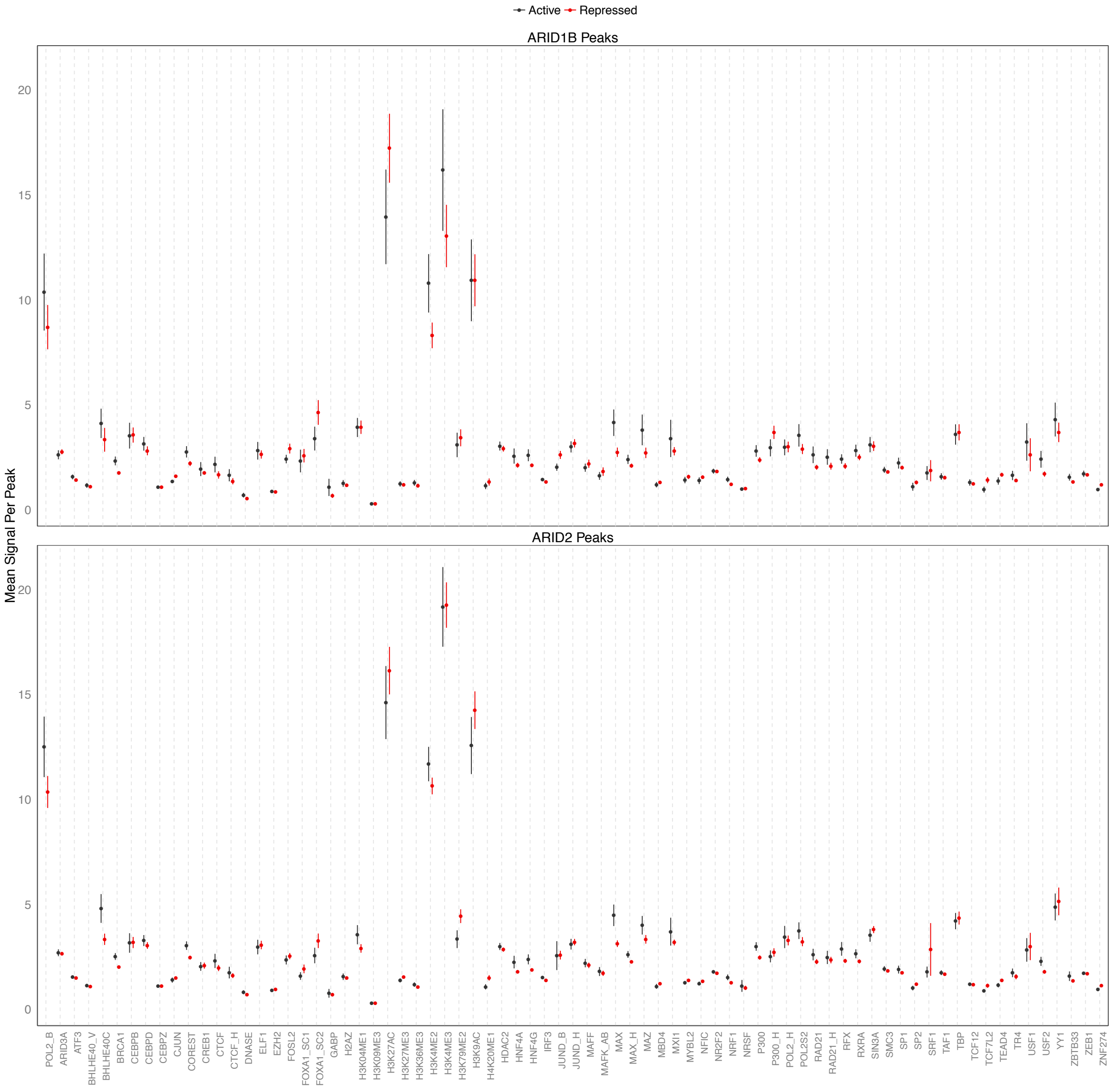

Supplmental Fig. 13: Enrichment of transcription factors and histone modifications at peaks associated with activation or repression

These data underly the selection of genes in Figure 7C. We compared the mean signal values for histone modifications and transcription factors at ARID1B and ARID2 peaks associated with either activation or repression. These data were used to identify factors that were more enriched when associated with repressed compared to active regions or vice versa.

Supplemental References

[1] Dunham I, Kundaje A, Aldred SF, Collins PJ, Davis CA, Doyle F, et al. An integrated encyclopedia of DNA elements in the human genome. Nature. 2012 sep;489(7414):57–74. Available from: http://dx.doi.org/10.1038/nature11247.

[2] Euskirchen GM, Auerbach RK, Davidov E, Gianoulis TA, Zhong G, Rozowsky J, et al. Diverse Roles and Interactions of the SWI/SNF Chromatin Remodeling Complex Revealed Using Global Approaches. PLoS Genetics. 2011 mar;7(3):e1002008. Available from: http://dx.doi.org/10.1371/journal.pgen.1002008.

[3] Ernst J, Kheradpour P, Mikkelsen TS, Shoresh N, Ward LD, Epstein CB, et al. Mapping and analysis of chromatin state dynamics in nine human cell types. Nature. 2011 mar;473(7345):43–49. Available from: http://dx.doi.org/10.1038/nature09906.
